# Machine Learning Analysis of Electronic Nose in a Transdiagnostic Community Sample With a Streamlined Data Collection Approach: No Links Between Volatile Organic Compounds and Psychiatric Symptoms

**DOI:** 10.3389/fpsyt.2020.503248

**Published:** 2020-09-16

**Authors:** Bohan Xu, Mahdi Moradi, Rayus Kuplicki, Jennifer L. Stewart, Brett McKinney, Sandip Sen, Martin P. Paulus

**Affiliations:** ^1^ Laureate Institute for Brain Research, Tulsa, OK, United States; ^2^ Department of Computer Science, Tandy School of Computer Science, University of Tulsa, Tulsa, OK, United States; ^3^ Department of Community Medicine, Oxley College of Health Sciences, University of Tulsa, Tulsa, OK, United States; ^4^ Department of Mathematics, College of Engineering & Natural Sciences, University of Tulsa, Tulsa, OK, United States; ^5^ Department of Psychiatry, School of Medicine, University of California San Diego, San Diego, CA, United States

**Keywords:** machine learning, data mining, electronic nose, mental health, exhalomes, computational psychiatry

## Abstract

Non-intrusive, easy-to-use and pragmatic collection of biological processes is warranted to evaluate potential biomarkers of psychiatric symptoms. Prior work with relatively modest sample sizes suggests that under highly-controlled sampling conditions, volatile organic compounds extracted from the human breath (exhalome), often measured by an electronic nose (“e-nose”), may be related to physical and mental health. The present study utilized a streamlined data collection approach and attempted to replicate and extend prior e-nose links to mental health in a standard research setting within large transdiagnostic community dataset (N = 1207; 746 females; 18–61 years) who completed a screening visit at the Laureate Institute for Brain Research between 07/2016 and 05/2018. Factor analysis was used to obtain latent exhalome variables, and machine learning approaches were employed using these latent variables to predict three types of symptoms independent of each other (depression, anxiety, and substance use disorder) within separate training and a test sets. After adjusting for age, gender, body mass index, and smoking status, the best fitting algorithm produced by the training set accounted for nearly 0% of the test set’s variance. In each case the standard error included the zero line, indicating that models were not predictive of clinical symptoms. Although some sample variance was predicted, findings did not generalize to out-of-sample data. Based on these findings, we conclude that the exhalome, as measured by the e-nose within a less-controlled environment than previously reported, is not able to provide clinically useful assessments of current depression, anxiety or substance use severity.

## Introduction

Volatomics, the study of volatile metabolites (1) (such as ethanol and amino acids) and emanations from the human body, especially human breath known as exhalome, is an area of active investigation (2). The human exhalome contains more than 1,800 volatile organic compounds (VOCs) (3), which researchers hope can elucidate the inner workings and status of bodily functions. This hope for exhalome as a measure of physical and mental health status is well-grounded as a growing literature links various types of psychopathology with altered central and peripheral markers of bodily processing within the bidirectional brain–body context (4–7). Few exhalome studies have examined the extent to which VOCs are altered as a function of psychopathology and investigations such as the present study could help fill this gap in the field.

Some have argued that the brain itself is an endocrine gland that triggers stress responses (8). VOC patterns in the breath may shed light on brain–body dysfunction (9). For example, upper respiratory tract infections such as influenza are linked to stress (10) that could be associated with dysfunctional changes in exhaled breath composition. Moreover, impaired quality of life (11) and presence of obsessive compulsive and bipolar disorders (12) are also linked to upper respiratory tract infections. More research is warranted to investigate the utility of exhalome for indexing clinical symptoms and potentially, differentiating between particular psychiatric disorders.

In psychiatry, assessment of illness severity relies almost entirely on report by the affected individual or by a mental health provider, which can be subject to a number of different biases. The identification of quantitative measures of illness severity, which is based on the underlying biological processes that are affected by the disorder, would be a major advance in the field. The measures could have broad applicability for the selection of individuals for treatment and the monitoring of the efficacy of different behavioral or pharmacological interventions. Moreover, these types of measures would build a much stronger case for hypothesis testing and reaching objective, evidence-based conclusions (13). As many clinical symptoms are transdiagnostic, or present across multiple mental disorders (*e.g.*, insomnia, appetite change, negative mood, concentration difficulties), identifying non-invasive biological markers of dimensional as well as categorical symptom clusters could improve mental health screening and intervention efforts (14).

Modern breath analysis can be traced back to the seminal work of Pauling et al. (15) wherein they showed the presence of a colorful cast of compounds in human breath, using gas–liquid partition chromatography (16). Two classes of instruments have traditionally been used for breath analysis: (1) gas chromatographic technologies coupled with a mass spectrometric detector (GC–MS); and (2) electronic “nose” also known as “e-nose” (17). Early studies (18) used GC–MS technologies that are relatively expensive, difficult to use, and require specially-trained and field-experienced technicians to operate (19). Over the past two decades, exhalome researchers have increasingly employed easy-to-use, non-invasive, relatively fast, and low-cost tools (16) to investigate links between exhalome and symptoms of physical and mental health disorders (12, 13, 19). In contrast to GC–MS technology, e-nose devices enable researchers to easily study “smell-prints” (the molecular pattern of chemical compounds recorded by the sensors inside the device) derived from various VOCs using pattern recognition and modern machine learning methods (16).

E-nose-driven metrics have shown potential in differentiating case/control groups, especially in respiratory diseases (16). For example, Dragonieri et al. (20) distinguished between controls and people with asthma (with 10 subjects per group, four different groups), without the need to observe intricate molecular components of the breath. One of the main differences between GC–MS and an e-nose is that e-nose researchers do not need to confront a long list of compounds and their concentrations within a particular sample, but instead need to know to what degree a detected smell-print matches a known compound pattern (21). From an evolutionary biology perspective, this process is roughly similar to the way the human olfactory system has evolved since it does not directly recognize the presence of a particular chemical compound; rather, it senses a pattern similar to what it has already been experienced by the brain without knowing what particular chemical compound has implemented that pattern on the sensory organ (17, 22). The study of human breath, as well as potential biomarkers it may actualize to monitor brain health and functions, has not been fully investigated within large samples using robust statistical methods (13). Additional research in this area could pave the way for the establishment of breath analysis in the diagnosis of various psychiatric symptoms.

According to a recent review (22), biological sources of the VOCs in respiration measured by e-nose devices are known to some extent. In the work of Bajtarevic et al. (23), isoprene, acetone, methanol, and benzene were employed as biomarkers of lung cancer. The concentrations of these VOCs decreased in patients compared to healthy subjects due to uncontrolled creation of new and unnecessary lung cells as well as retention of old damaged cells (24). However, Sánchez et al. (22) also noted that the VOCs present in exhaled breath are not necessarily produced by endogenous biochemical processes (*e.g.*, acetonitrile is commonly found in the breath of smokers, occurring exogenously).

Breath analysis has shown some success in indexing a variety of physiological symptoms (13, 17) within modestly sized samples, demonstrating that candidate VOCs can plausibly index the presence of certain disorders within individuals. For instance, e-nose technology has distinguished between: (1) smokers and non-smokers (21); (2) mild/severe asthmatics from non-asthmatics (20); (3) smokers with and without lung cancer (25); and (4) individuals with Alzheimer’s disease, Parkinson’s disease, and healthy controls (26, 27). Moreover, altered levels of nitric oxide in the breath have been associated with: (1) cardiovascular, neurological, and respiratory disorders (13); and (2) increased negative affect (anxiety, depression) and stress *via* weakening of the immune system (28–33). Further research is warranted to determine whether a broader spectrum of VOCs beyond nitric oxide is implicated in psychiatric symptoms (13).

There has been attempts to standardize e-nose instruments and sampling procedures and highlight the potential technical issues for exhalome research (34). However, there are some gaps to be filled in the exhalome literature, including, small and non-representative sample sizes and failure to account for non-linearity of data arising from the measurement of exhaled breath (13, 35). In addition, variability in e-nose detectors (whether commercially available or custom-built in labs), which are typically constructed with a small number of sensor arrays (14) could limit the resolution to detect complex VOC patterns in breath samples. Although the recommended e-nose analysis pipeline for breath analysis consists of “data acquisition, data pre-processing including data reduction/feature selection, generation of a pattern recognition algorithm in a training set, and testing of the algorithm in a validation set” (36), some studies do not perform external validation or confirmation of their findings, thereby limiting reliability and validity of their reports (37).

In investigations where a cohort design is analyzed and individuals with physical and/or mental disorder comorbidities will be involved, it is postulated that conventional unsupervised methods like principal component analysis (PCA), which has been widely used thus far in exhalome studies (16), will have difficulty differentiating between cases and controls (37). Although researchers recommend that supervised dimension reduction techniques such as partial least squares discriminant analysis (PLS-DA) be used in such designs, the combination of PCA and linear discriminant analysis (LDA) tends to yield more consistent results (36). Furthermore, with respect to clinically relevant prediction/classification, no published exhalome studies have employed a substantial heterogeneous sample of individuals to identify whether exhaled breath patterns can differentiate transdiagnostic clinical symptoms (*e.g.*, negative affect, anxiety, substance use, and depression). In order for a particular VOC pattern to be a useful biomarker of impairment, it must be sensitive and specific, distinguishing abnormal from normal functioning. In addition to data analysis concerns, issues regarding the collection of e-nose data are crucial to address. For e-nose to be more widely tested in research settings, hardware must be easy and straightforward to use and validated in less-controlled environments (*e.g.*, outpatient clinic or hospital setting).

The goal of this study was to identify whether patterns of human exhalome collected with a straightforward sampling approach and extracted by modern instruments (e-nose) and analyzed by machine learning approaches can replicate prior work linking VOC patterns to depression and anxiety symptoms (38). As some research has attempted to show that breath patterns vary as a function of gender and age (39), we also incorporated gender and age as factors in our analysis. As more women than men suffer from mood and anxiety disorders (40), breath patterns may show differential classification for men and women. To measure exact breath composition patterns, we utilized an e-nose (Cyranose 320; Smiths Detection, Pasadena, CA, USA) with 32 sensors to improve VOC detection accuracy and reliability (21). Furthermore, we investigated whether these goals are achievable under less-controlled, simpler sampling conditions without the need for sophisticated equipment such as VOC filters, separated air tubes, valves, medical air capsules and controlled sampling room conditions.

## Methods

### Participants

A total of 1,550 participants (947 female; ages 18–66 years) were recruited *via* fliers, radio, and internet advertisements from the greater Tulsa, OK area and completed a screening visit at Laureate Institute for Brain Research (LIBR) between 07/01/2016 and 05/21/2018 to determine further eligibility for various ongoing studies at LIBR. Participants with psychosis or cognitive impairments or medical conditions causing neuropsychiatric disorders were excluded. Written and informed consent was obtained from all participants, and the study was approved by Western IRB, WIRB Protocol No. 20101611. Participants received compensation for their participation.

**Figure 1:**
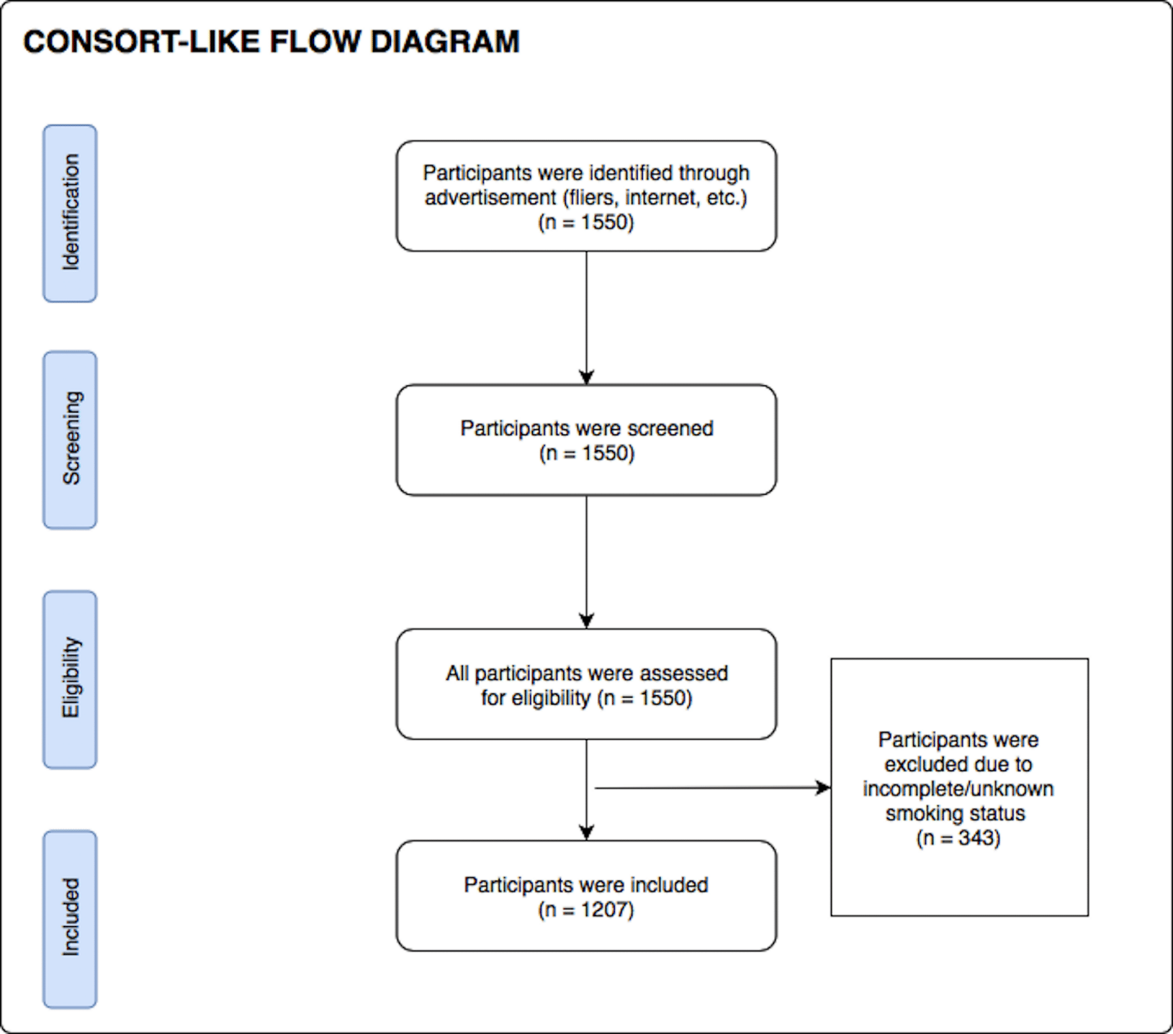
Consort-like diagram for participant inclusion, according to research inclusion criteria.

During their screening visit, participants completed a demographics questionnaire (to obtain age, gender, and nicotine smoking status) as well as the Patient Health Questionnaire 9 (PHQ-9) (41), the Drug Abuse Screening Test (DAST-10) (42, 43) and the Overall Anxiety Severity and Impairment Scale (OASIS) (44) to index symptoms of depression, substance use disorder, and anxiety respectively. Body mass index (BMI) was calculated by using an InBody370 Impedance Body Composition Analyzer (InBody Co., Ltd., South Korea). After excluding participants with incomplete/unknown smoking status data, 1,207 participants were included in the analysis. Although formal sample size estimation was not performed prior to study start, a sample of 1,207 subjects was sufficient to detect an effect with Cohen’s d of 0.081 with 80% power and significance level of 0.05. When queried about their nicotine use status, 36% (*n* = 435) were found to be current smokers and 64% (*n* = 772) were found to be non-smokers. The consort diagram for participant inclusion in this work is presented in **Figure 1**. Demographic and clinical characteristics of the final participants involved in this study are presented in **Table 1**. All participants were instructed to abstain from any food, drink, and chewing gum consumption, except for water, within 2 hrs of breath sample collection, and refrain from smoking and brushing their teeth.

**Table 1:** Participant characteristics.

	Overall(n = 1207)
**Age**	
Mean (SD^a^)	32.1 (10.4)
Median [Min, Max]	30.0 [18.0, 61.0]
Missing	4 (0.3%)
**Sex**	
Female	756 (62.6%)
Male	451 (37.4%)
**Smoking Status**	
Current Smoker	435 (36.0%)
Non-Smoker	772 (64.0%)
**BMI^b^**	
Mean (SD^a^)	27.6 (5.77)
Median [Min, Max]	26.6 [16.1, 52.1]
Missing	21 (1.7%)
**Depression PHQ-9^c^**	
Mean (SD^a^)	9.29 (7.15)
Median [Min, Max]	8.00 [0.00, 27.0]
Missing	19 (1.6%)
**Anxiety OASIS^d^**	
Mean (SD^a^)	7.05 (4.92)
Median [Min, Max]	7.00 [0.00, 20.0]
Missing	20 (1.7%)
**Addiction DAST-10^e^**	
Mean (SD^a^)	1.74 (3.01)
Median [Min, Max]	0.00 [0.00, 10.0]
Missing	18 (1.5%)
**Race Ethnicity**	
White	723 (59.9%)
Black	129 (10.7%)
Native American	190 (15.7%)
Hispanic	66 (5.5%)
Asian	22 (1.8%)
Other	47 (3.9%)
Missing	30 (2.5%)
**Education**	
Less than seven years of school	2 (0.2%)
Junior high school (7th, 8th, 9th)	22 (1.8%)
Some high school (10th, 11th)	55 (4.6%)
High school graduate (including equivalency exam)	216 (17.9%)
Some college or technical school (at least one year)	488 (40.4%)
College graduate	282 (23.4%)
Graduate professional training (Masters or above)	92 (7.6%)
Other	11 (0.9%)
Missing	39 (3.2%)

^a^Standard deviation.

^b^Body mass index.

^c^Patient Health Questionnaire 9.

^d^Overall Anxiety Severity and Impairment Scale.

^e^Drug Abuse Screening Test 10.

### Technology and Hardware

A commercially available e-nose (Cyranose 320; Smiths Detection, Pasadena, CA, USA), was utilized to acquire exhaled VOC patterns (sampling procedure below). This e-nose utilizes 32 sensors and on-board pattern recognition algorithms to detect chemical vapors of interest to produce a “smell print”. As these sensors are semi-selective for various compounds, all of them will respond to the mixture (breath sample) in varying degrees. Sample differentiation is organized by basic properties (polarity, hydrogen bonding, acidity/basicity, *etc.*) rather than by specific compounds (*e.g.*, oxygen, nitric oxide, carbon dioxide) meaning that it seeks an established pattern of compounds on the sensor array and not each specific chemical compound alone. First the e-nose is initiated by registering levels of ambient air (baseline measurement) over time. Next, when VOCs from exhaled breath pass over organic insulated polymer within the e-nose, polymer swelling produces a change in electrical resistance output as a function of time for each sensor. Raw signals are output as a function of resistance (ohms) by time (seconds) for baseline and registered breath. These signals are then processed to compute percent signal change from baseline for each of the 32 sensors.

### Sampling Procedure

After participants provided informed consent and completed questionnaires, they provided one exhaled breath sample, collected in sampling bags comprised of Nalophane film (16 in) and a 3-in poly-tetra-fluoro-ethylene tube attached as a mouth-piece for the participant (and also as an interface to be attached to the e-nose). Nalophane bags were employed, because they are cost-effective, reliable and well-suited for exhalome investigations (45). The breath sample collection procedure was administered by a trained research assistant. Participants were instructed to take a deep breath and blow one vital capacity exhalation into a sampling bag. A baseline metric of ambient air from the e-nose equipment (6 min) was obtained in accordance to manufacturer’s guidelines. The bag was secured and connected to the e-nose to sample the exhaled breath for duration of 1 min. This procedure was followed by a subsequent ambient air collection (1 min) and then the second breath sample measurement was performed for another 1 min from the same sampling bag in the same manner described above.

### Data Preprocessing

First, raw e-nose breath signals were corrected for baseline drift (25) by fitting a five-degree polynomial to the signal acquired from each of the 32 e-nose sensors. Second, percent signal change (PSC) from baseline was computed as the corrected breath signal divided by the corrected baseline signal, with the highest value during the first breath sample as the metric of interest for each of the 32 sensors. Third, a regression model was fitted using an 11th-order polynomial with respect to each participant’s e-nose collection date to correct long-term temporal drift (36). Fourth, to correct for heterogeneity in overall VOC concentration magnitude across participants, a double standardization procedure was performed on the data: (1) all participants’ breath samples were standardized (z-scored) on one sensor; and (2) all 32 sensor responses were standardized on one participant’s breath sample. Fifth, as several sensor responses were significantly correlated, principal components analysis (PCA) was applied as an exploratory machine learning method to reduce data dimensions and generate linearly independent breath factors. All 32 principal components were used for data analysis. Details regarding data preprocessing steps are presented in the **Supplementary Material**.

### Statistical Analysis

We computed intraclass correlations (ICCs) (46) based on responses from both breath sample draws for each sensor to evaluate the short-term stability of sensor readings. Large ICCs would indicate relatively stable measurements, implying little difference between selecting the first or second sample draw. Conversely, small ICCs would be indicative of rapidly decaying or unstable sensor measurements.

**Figure 2:**
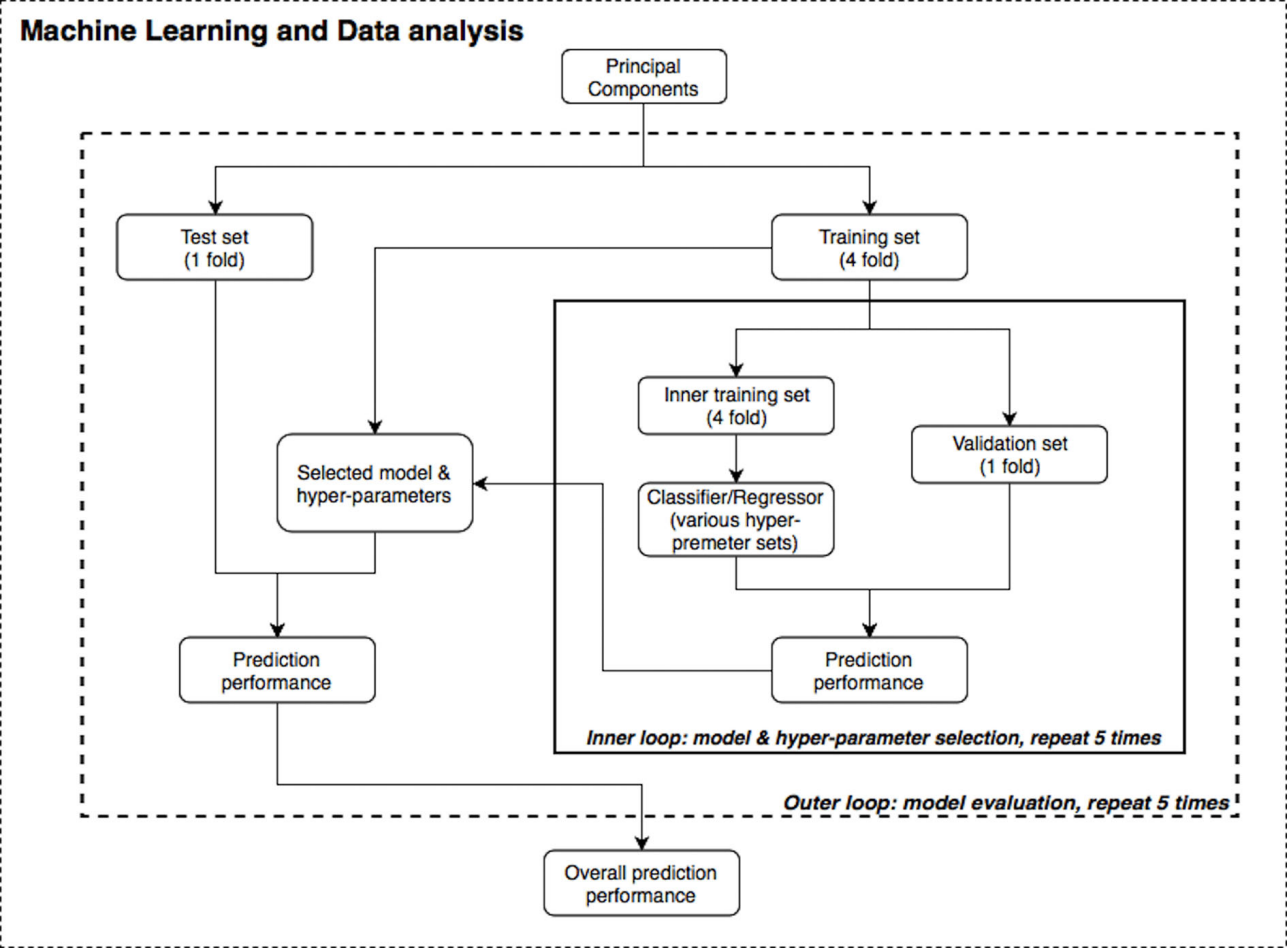
Statistical data analysis and machine learning pipeline.

**Figure 2** illustrates the machine learning analysis pipeline used in the present study, wherein transformed PSC e-nose data were related to demographic and clinical variables. In machine learning, it is common to perform cross-validation so that a model is repeatedly trained and tested on the dataset to obtain robust performance and accuracy results. A training set is used in order to let the machine “learn” from the data (fit the model to data) and a test set is used for evaluating the fitted model on training data in terms of accuracy (how close the model’s output is to the real data).

Supervised machine learning algorithms were applied to the transformed PSC data using the R and Python statistics platforms. Specifically, Random Forest (RF) (47–49), Support Vector Machine (SVM) (50), and linear/logistic regression learning algorithms with varying hyperparameters and nested cross-validation (51, 52) (five-fold for both the inner and outer loops) were applied to the questionnaire, BMI, and e-nose data. SVM solves an optimization problem to find support vectors which are a subset of points from the training dataset, and the decision boundary is calculated based on these support vectors. On the other hand, RF is an ensemble learning method that is constructed by multiple bagged decision trees. To evaluate model accuracy, Area Under the receiver operating characteristic Curve (AUC) and R^2^ values were used. The primary variables of interest related to e-nose VOCs were three mental health variables: PHQ-9, OASIS and DAST-10 scores. Other measures included were age, sex, BMI, and nicotine smoking status. We also attempted to replicate the results from Cheng et al. (21), who differentiated subject smoking status based on the first two PCs.

Furthermore, nested validation was applied for model hyperparameter tuning and feature selection (*e.g.*, selecting number of trees in RF or regularization term in SVM) in each inner loop iteration (53). The dataset was first divided into five disjointed and equally-sized subsets or “folds”. There were two nested loops (inner and outer loops) within this pipeline (see **Figure 2**) and on each repetition, one of the folds was used as the test/validation set, whereas the remaining folds were treated as the training set. Although the divisions were established by randomization for regression problems (age, BMI and mental health scores), one-way analysis of variance (ANOVA) test was implemented to ensure these subsets had the same population mean of the dependent variable; stratified division was applied for classification problems (gender and smoking status). Both loops were iterated five times to evaluate and cross validate results. In each run of this nested CV structure, prediction performance was measured, and the model with the best accuracy was specified as the final result of this pipeline.

## Results

**Figure 3:**
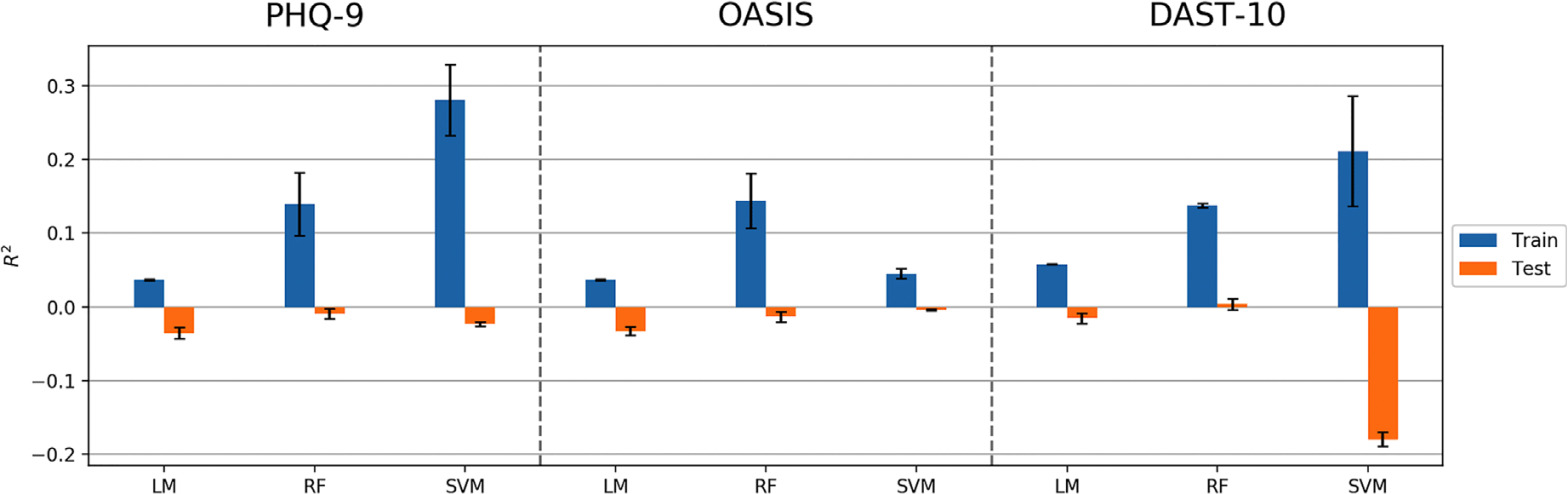
Model performance (R^2^ values) in predicting PHQ-9, OASIS, and DAST-10 using Linear Model, Random Forest (RF), and Support Vector Machine (SVM) algorithms. Error bars represent standard deviations of R^2^ values.

**Figure 4:**
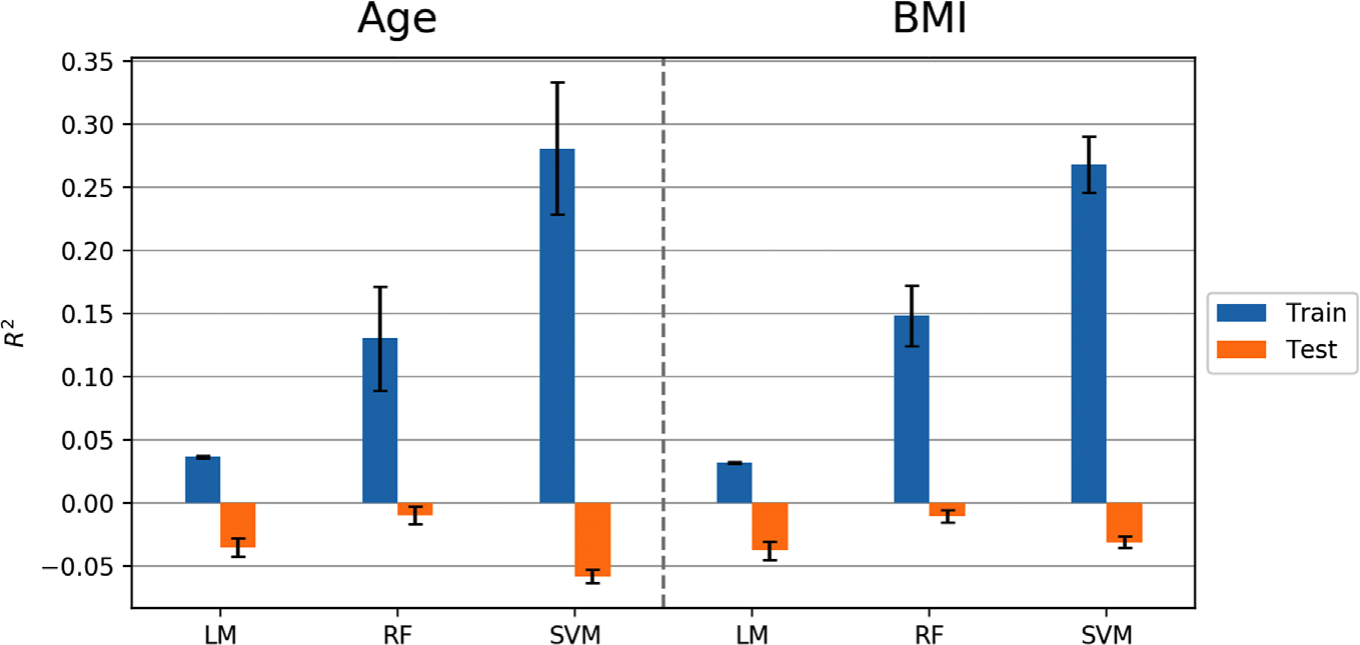
Model performance (R^2^ values) in predicting age and BMI using Linear Model, Random Forest (RF), and Support Vector Machine (SVM) algorithms. Error bars represent standard deviations of R^2^ values.

The machine learning pipeline (**Figure 2**) was applied to depression, anxiety, and addiction variables of interest including PHQ-9, OASIS, and DAST-10 (**Figure 3**). In general, although some of the algorithms learned to predict different psychiatric symptoms, explaining as much as 20% of the variance (blue bars, **Figure 3**), these models did not generalize to the test set (orange bars, **Figure 3**). For the independent test dataset, very little variance was accounted for, and in one case, the prediction was worse than just predicting the mean of the test sample (negative variance accounted for). **Supplementary Material** provides additional illustrations of machine learning analyses. This pattern of results is most consistent with model overfitting to the training dataset. The R^2^ value of the test dataset being smaller than or near 0 (orange bars, **Figure 3**) indicates that the fitted model is worse or not much better than the null hypothesis (a model that always predicts the mean value for any input). Models for age and BMI have similar prediction performance as those for mental health variables (**Figure 4**).

**Figure 5:**
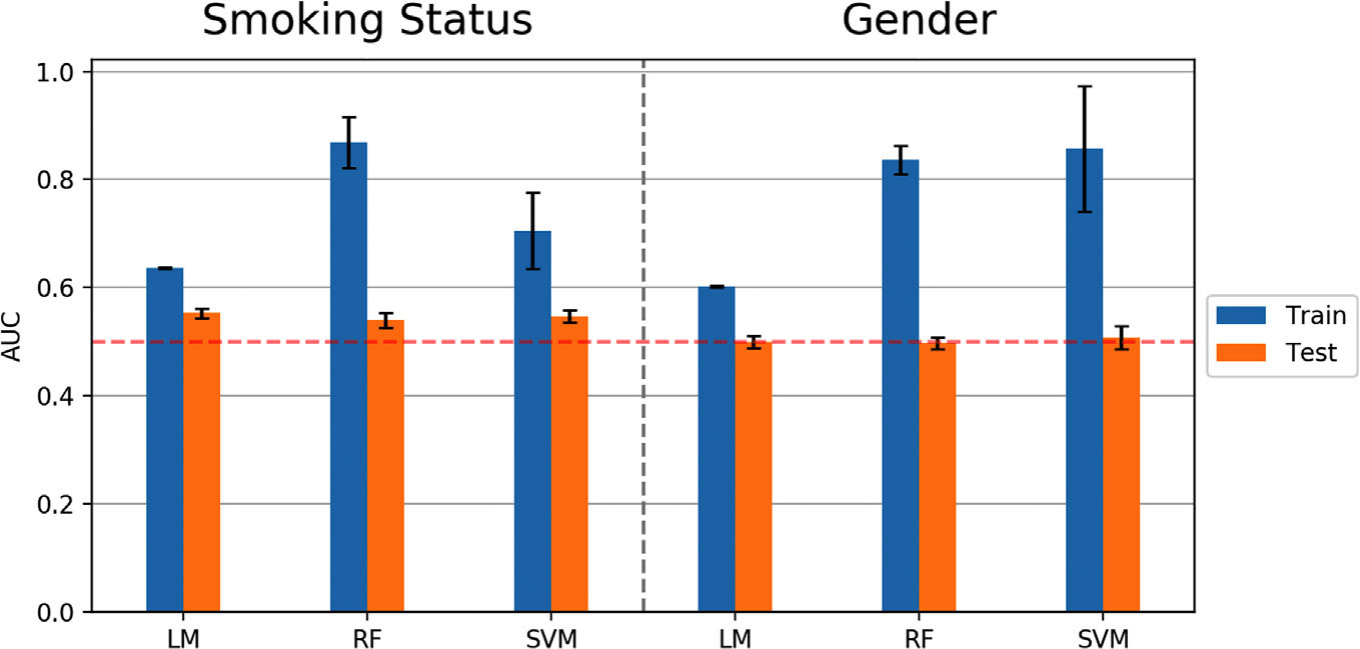
Model performance (AUC values) in predicting smoking status and gender using Linear Model, Random Forest (RF), and Support Vector Machine (SVM) algorithms. Error bars represent standard deviations of AUC values.

**Figure 6:**
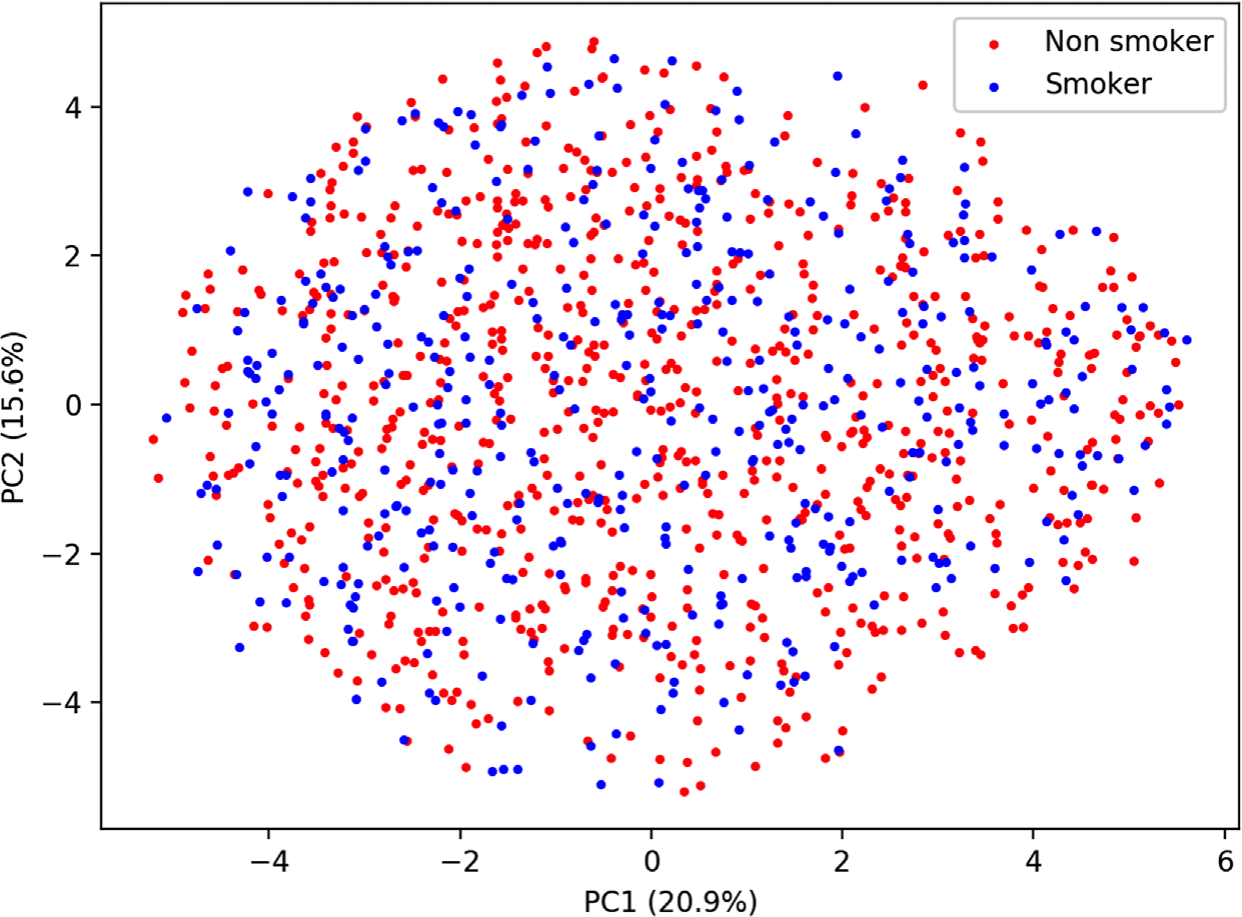
Principal component analysis plot of smoking status.

Similar to predicting continuous outcome variables, evidence for overfitting was observed when predicting dichotomous outcomes: smoking status and gender. Area under the curve (AUC) was used to measure model performance for predicting smoking status and gender. Cross-validated AUC in the training data (blue bars, **Figure 5**) was above 0.5 (red-dashed line) for all methods. However, the AUC on independent test data (orange bars, **Figure 5**) was consistent with 0.5 AUC, which is the null value of no discrimination capacity to distinguish between positive and negative classes. Our main focus was to assess the generalizability of machine learning models that use the sensor measurements directly (**Figures 3**–**5**). As a secondary analysis, we also attempted to reproduce the results from (21), which differentiated smoking status based on the first two PCs with highest variance. In our data, no cluster(s) were present to separate smokers from non-smokers in the two-dimensional PC space (**Figure 6**).

The ICC values indicated a strong reliability of e-nose measurements within a short period of time. The sensor value ICCs are between 0.91 and 0.99, with the exception of Sensor 8, which produced an ICC of 0.80. Together, these values show the stability (54) of sensor reading between selecting the first and second sample draws.

## Discussion

The current investigation employed multiple machine learning approaches on a sizable community sample of 1,207 individuals to further previous research linking e-nose exhalome to assessment of psychiatric symptom severity in less-controlled research settings. Although our results showed that machine learning algorithms using cross-validation were able to achieve accuracy and account for variance above the null hypothesis in the training sample, the models did not generalize to an independent test sample, which is evidence for overfitting. Thus, we found no generalizable relationship between e-nose factors or PCA factors and the mental health symptoms of depression, anxiety, or substance use.

The main strength of the current study over previous studies is the larger sample size. This is the largest e-nose study ever conducted on a psychiatric population; our current sample of over 1,200 participants is substantially larger than published exhalome research (typically less than 100 subjects) (36, 55). Ligor et al. (56) used solid phase microextraction-gas chromatography combined with mass spectrometry (SPME-GC/MS) analyses with 484 subjects for selecting potential lung cancer biomarkers, which was the next largest sample we identified. A second strength of our study is the application of multiple analysis pathways suggested previously by researchers in the field. We utilized a rigorous machine learning paradigm with an iterative nested cross-validation approach, which involved splitting the dataset into training and testing sets on each iteration of model building and evaluation processes. To assess overfitting, we included an independent dataset to test final models. Overfitting could be a main culprit in overlooking the possible presence of false positives in prior work (57). A third strength is our use of multiple psychiatric symptoms to attempt to identify e-nose metrics as novel biomarkers in assessing and predicting mental illness. Exclusive reliance on self-report and traditional methods for diagnosis, treatment and monitoring of psychiatric symptoms is a current challenge in the field. There is a need for simple measures to predict the severity of these symptoms and develop more accessible and non-invasive biomarkers for this purpose in medicine and psychiatry.

In addition to possible overfitting in previous smaller studies, the lack of replication in the current study could also be due to the “winner’s curse” phenomena that has been observed in association studies: early studies tend to report a result with a substantial effect size, which is less likely to be seen in subsequent replication studies *i.e.*, GWAS and epidemiological investigations. In the long run, regression toward the mean would be a more achievable result expected to be observed (58). Furthermore, the sampling method to collect breath samples practiced in this work was different compared to previous works: in contrast to applying sophisticated sampling tubes, filters and special valves to breath data collection, we employed an accessible sampling setup within a conventional research/medical facility setting. Another factor limiting replication could be the dimensional design of the present study as opposed to categorical group comparisons (cases *versus* controls) reported in prior works; it is possible that effects become larger when comparing extremes of a phenotype (*e.g.*, healthy control *versus* symptomatic patient).

A limitation of the current study is our use of e-nose hardware, which is cheaper, more easily measured, and more easily used biomarker, but is less accurate than gas chromatography and mass spectrometry (GC–MS) technologies (59, 60). Unlike GC–MS, which detects specific chemicals and molecules within the breath, e-nose detects patterns of chemical compositions detected over sensor arrays, performing “smell-print” recognition (20). A second limitation is the possible presence of psychiatric comorbidities among participants, which might have impacted detection of particular symptom(s). Third, this study was cross-sectional, with data collected at only one time point during a screening session; employing similar analysis strategies on longitudinal e-nose and symptom data may result in more effective prediction of future illness severity.

Another possible limitation was that the breath sample was collected from the mouth using a relatively simple procedure; it might be the case that having stricter control or collecting samples from alternate airways, *e.g.*, nasal passages, may yield different sets of smell prints and resulting outcomes might change. Although some previous studies used more sophisticated devices or VOC-filtered room air for breath sample collection control (20, 21, 25), this study focused on investigating an easy, quick, and relatively inexpensive approach to evaluate mental health status. Another potential limitation of the breath sampling procedures in the present study might be when participants hold their breath. The breath-hold process involves anatomic dead space (61), and it has been shown that exhalation rate and breath-hold affect the levels of exhaled VOCs detected by the Cyranose 320 (62). Furthermore, although the effect of diurnal variations on the breath sample VOCs detected by e-nose has been investigated (63, 64), these variations were not observed in our pipeline (**Figure. S14** in **Supplement**).

The present study did not replicate prior studies linking e-nose breath metrics to mental health variables within the context of a less-controlled sampling environment than the GC–MS (21, 25). Given the limitations of this study, more work is needed to investigate whether e-nose technologies utilizing higher resolution sensor arrays and more sensitive materials can aid in the development of novel biomarkers to track psychiatric symptom severity.

## Data Availability Statement

The datasets generated for this study are available on request to the corresponding author.

## Ethics Statement

The studies involving human participants were reviewed and approved by the Western Institutional Review Board. The patients/participants provided their written informed consent to participate in this study.

## Author Contributions

MP developed the research question and conducted the research and supervised the project, as well as revised and edited the manuscript. BX and MM as joint first co-authors, performed the research, wrote the manuscript, and revised it. RK is the machine learning expert and supervised the data analysis pipeline and revised the manuscript. JS is field expert and revised the manuscript. BM is machine learning expert and revised the manuscript. SS revised the manuscript and gave feedback on data analysis. All authors contributed to the article and approved the submitted version.

## Funding

This research was supported by the Laureate Institute for Brain Research and the National Institute of General Medical Sciences (P20GM121312, MP, RK).

## Conflict of Interest

MP is an advisor to Spring Care, Inc., a behavioral health startup. He has received royalties for an article about methamphetamine in UpToDate. The author is supported by a grant from the National Institute of Mental Health (R01 MH101453), from the National Institute on Drug Abuse (U01 DA041089).

The remaining authors declare that the research was conducted in the absence of any commercial or financial relationships that could be construed as a potential conflict of interest.
