# Supplementary material for: Machine Learning Analysis of Electronic Nose in a Transdiagnostic Community Sample With a Streamlined Data Collection Approach: No Links Between Volatile Organic Compounds and Psychiatric Symptoms
Source: Front Psychiatry. 2020 Sep 16;11:503248. doi: 10.3389/fpsyt.2020.503248 (PMC7524957; doi:10.3389/fpsyt.2020.503248)
Supplement: Supplementary file 1 [file DataSheet_1.docx]

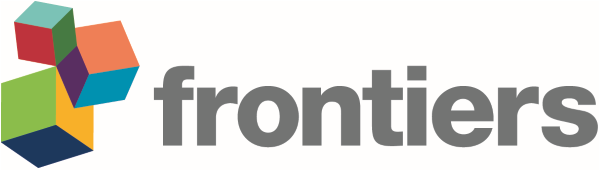


***Supplementary Material***

# SAMPLE COLLECTION BAG


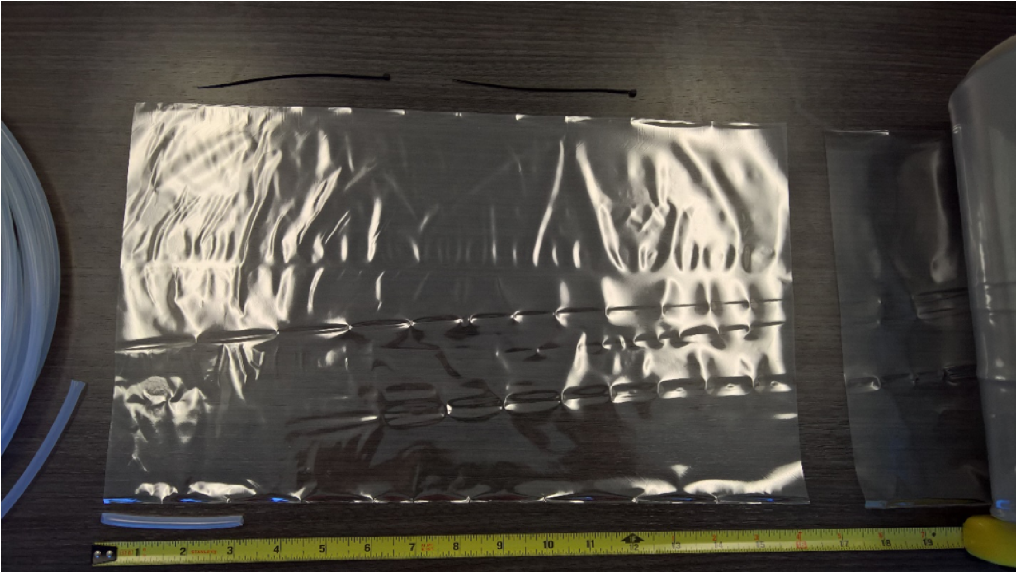

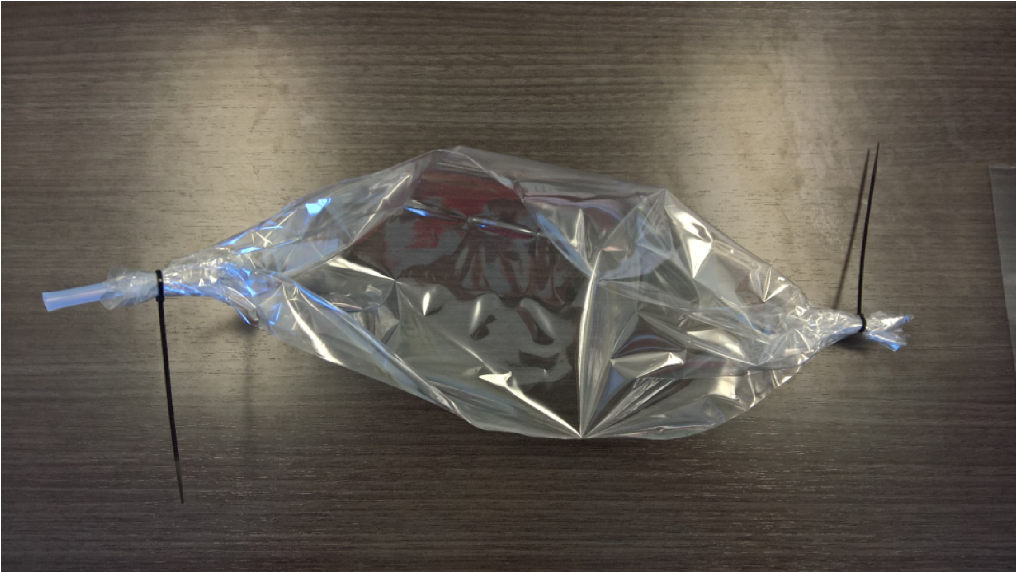


(A) Nalophan Film (B) Sampling Bag

Figure S1. Sample Collection Bag.

# RAW SENSOR OUTPUT


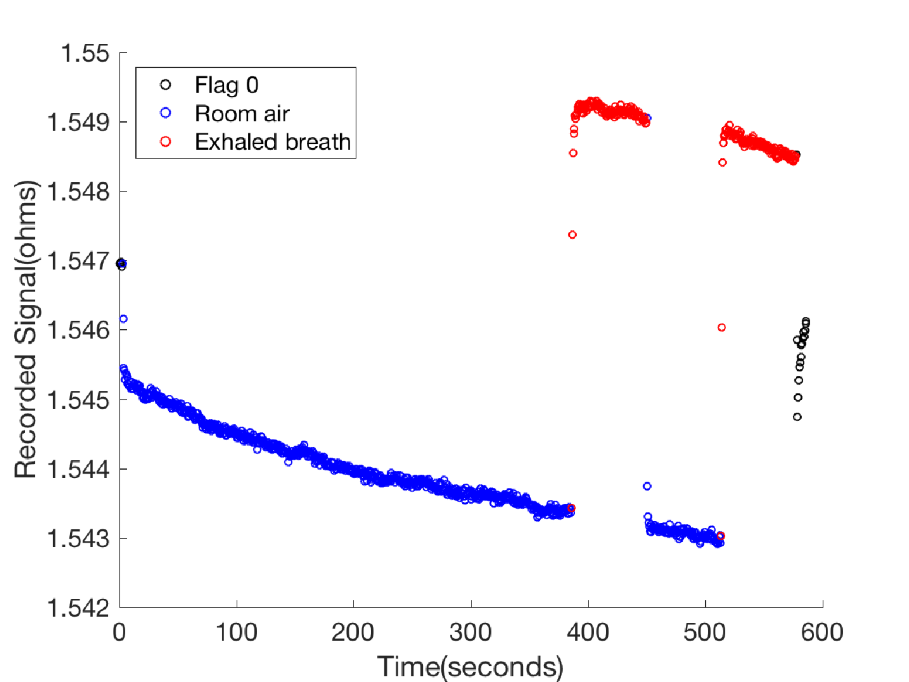

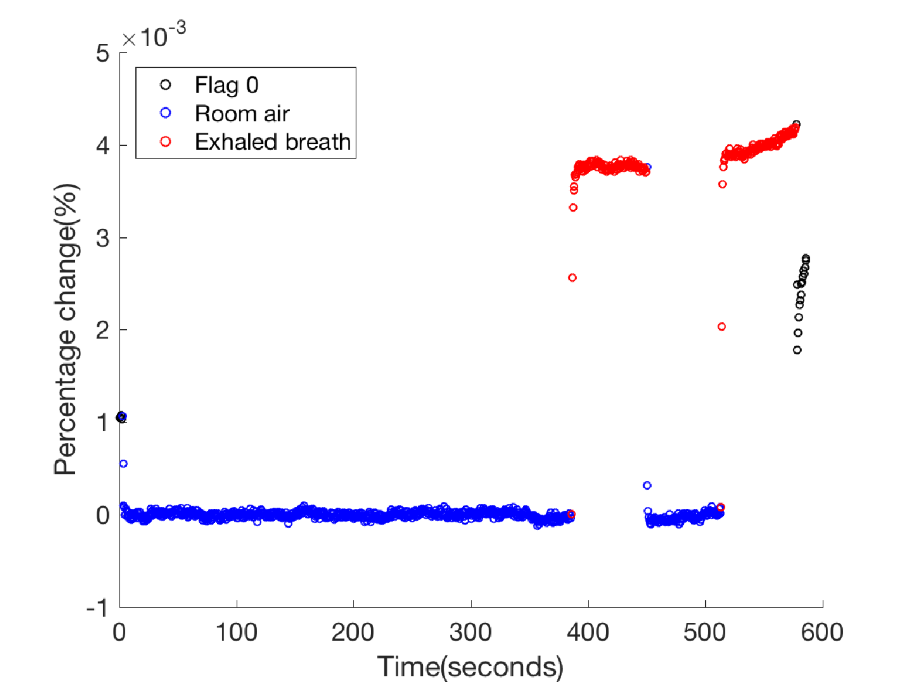


(A) Raw Sensor Output (B) Pre-processed Signal


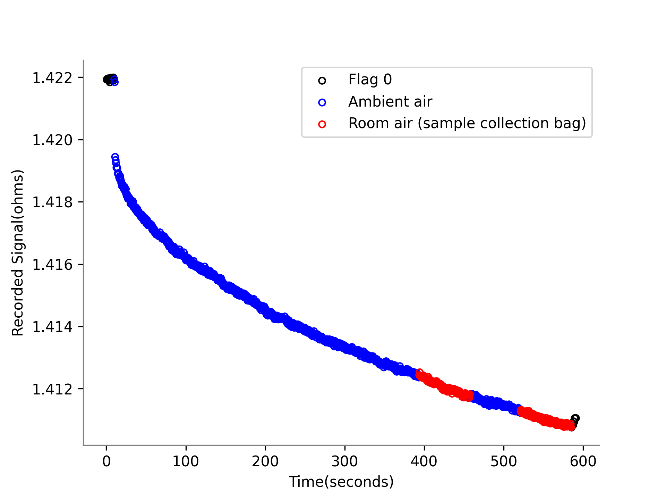


(C) Sample Collection Bag Noise

Figure S2. Typical response for one sensor. (A) A typical raw sensor response; (B) Percentage signal change after baseline shift correction; (C) Raw signal of Sensor 1 response to the ambient air in the room (blue) and ambient air collected in a sampling bag (red), the sample collection bag does not introduce background noise.

# LONG-TERM TEMPORAL DRIFT

Polymer oxidation may result in long-term drift (Bikov et al., 2015; James et al., 2005). The two

“humps” in Fig. S3(A) correspond to the data collected around January 2017 and 2018. However, from our inquiry from the manufacturer, the reason for this drift is not understood.


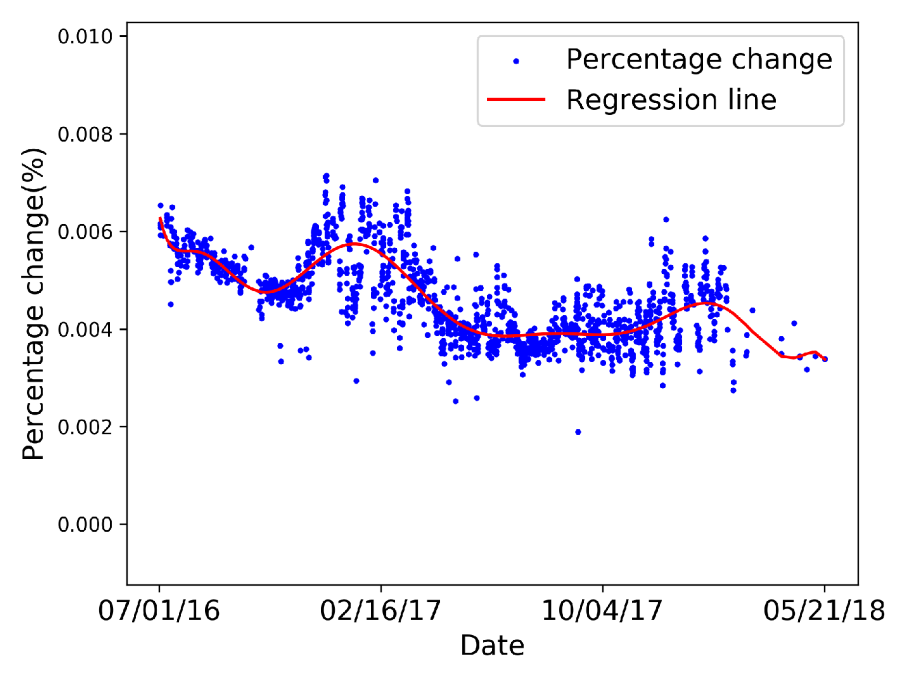

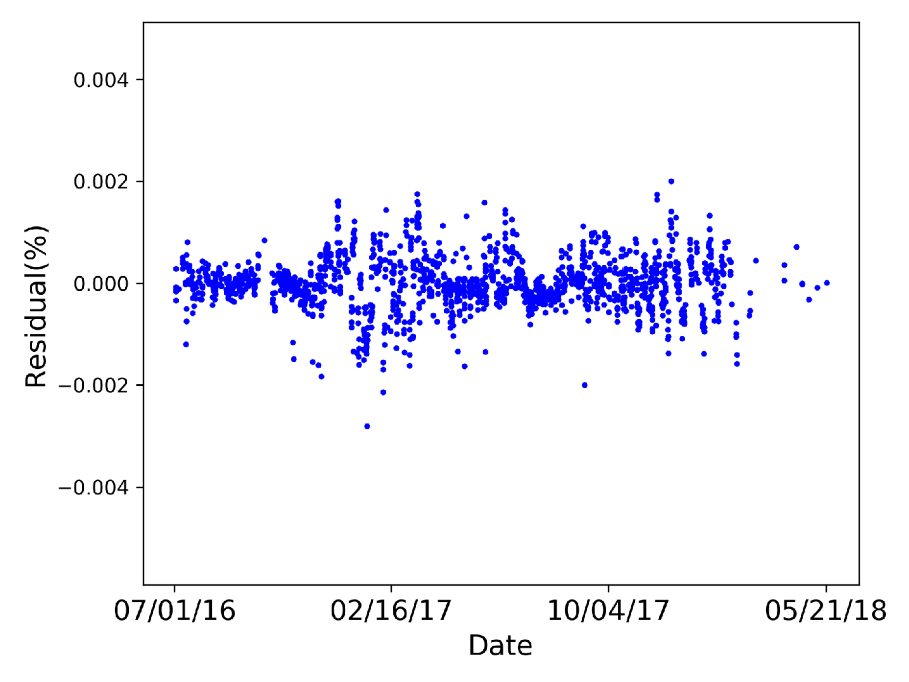


(A) Long-Term Temporal Drift (B) Long-Term Drift Correction

Figure S3. (A) A typical long-term temporal drift for one sensor, where each blue dot represents the highest percentage signal change value during the first breath sample measurement for each subject, and the red line represents an 11th-order polynomial fitted to blue dots with respect to collection date; (B) Residual values of percentage signal change.

# DOUBLE STANDARDIZATION

Figs. S4(A) and S5(A) plot the representative values and corresponding residuals (after long-term temporal drift correction) of all subjects across all sensors. The subject marked by “x” has low values and residuals from all sensors. One possible explanation for this participant’s low values is that the subject has lower overall VOC concentrations. On the other hand, the low overall VOC concentration may be a symptom of an illness. The double standardization is implemented since the relative difference between sensor responses of one subject may be more informative, and it eliminates the potential negative impact of low overall VOC concentrations.


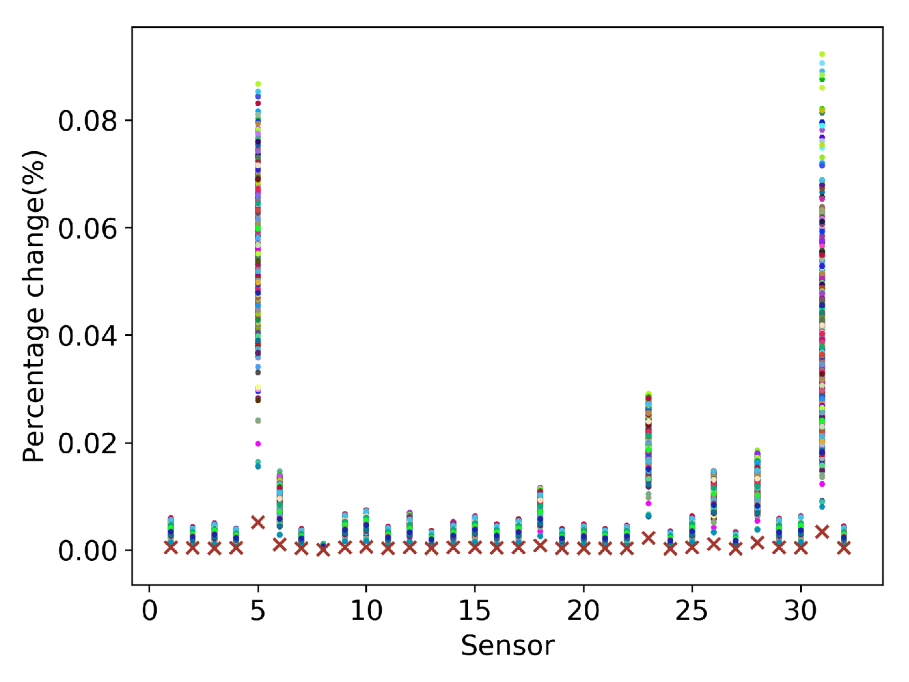

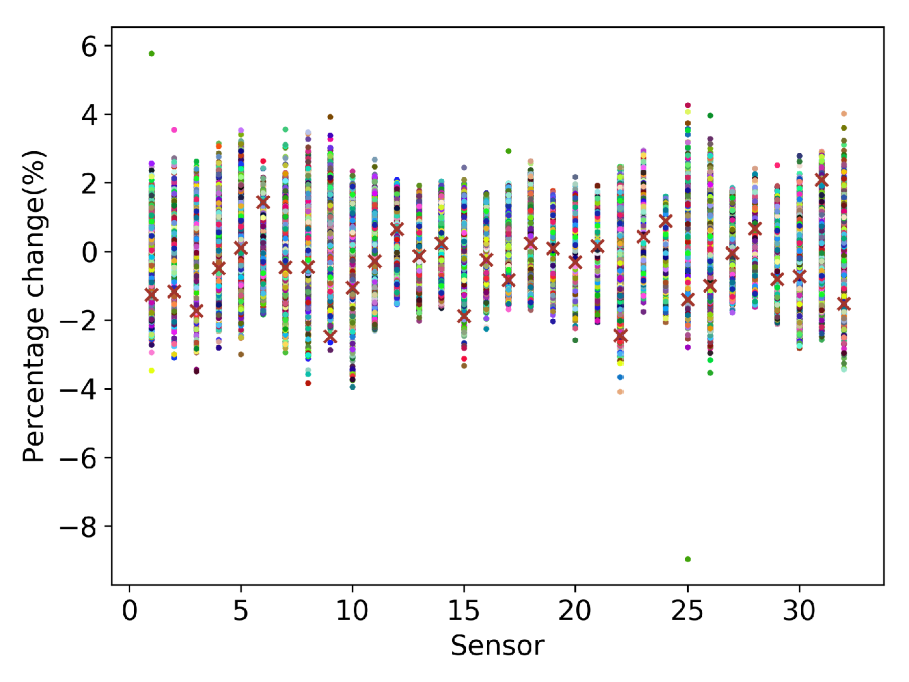


(A) Raw Data (B) Double Standardized Data

Figure S4. (A) Representative values for all subjects across all 32 sensors without long-term temporal drift correction, where one color represents one subject, the subject marked as “x” has relatively low values from all sensors; (B) Data after double standardization.


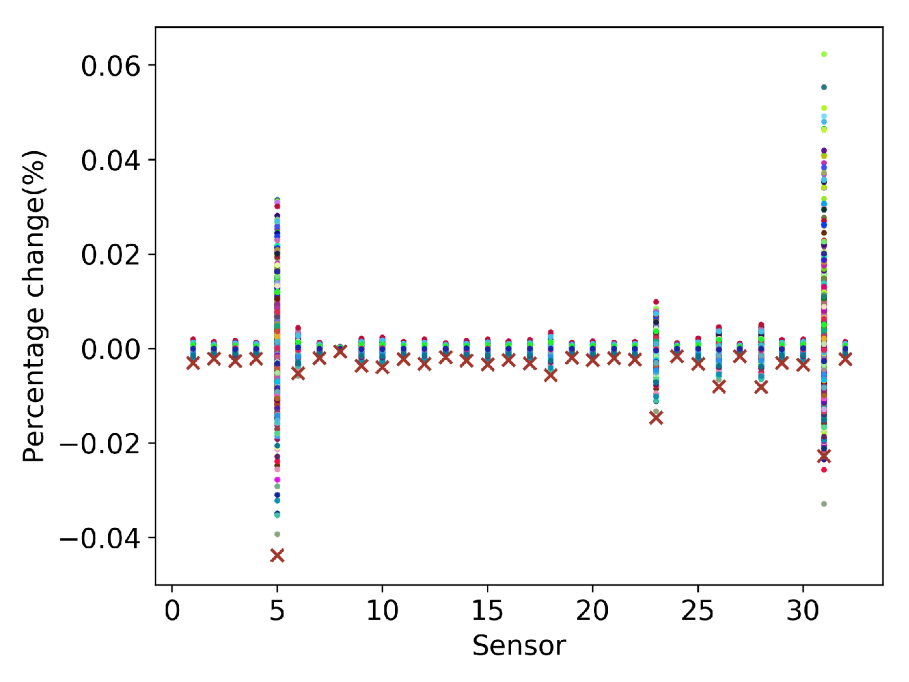

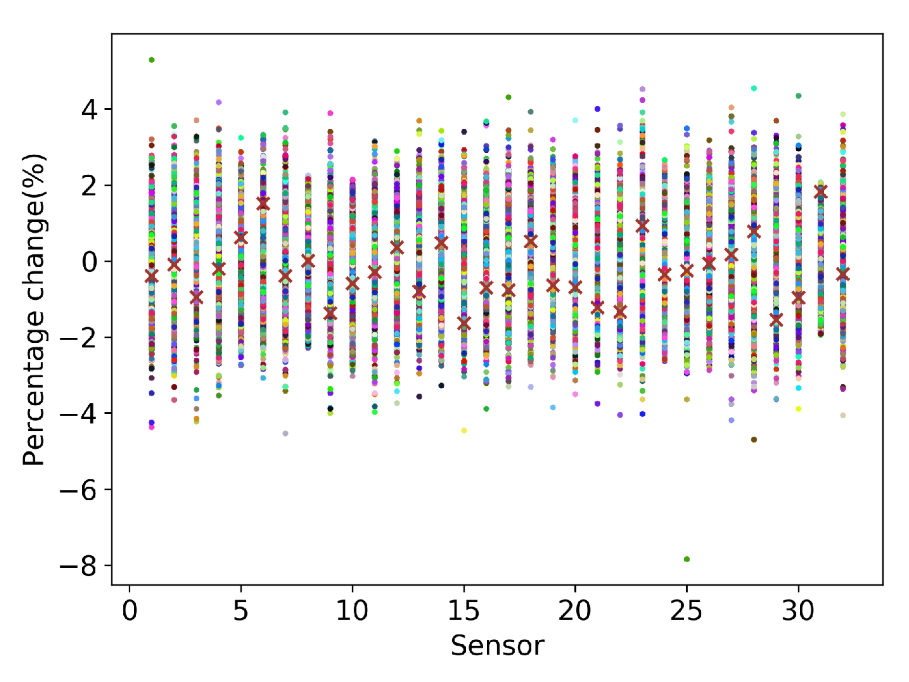


(A) Raw Residual (B) Double Standardized Residual

Figure S5. (A) Residual of representative values for all subjects across all sensors; (B) Residual after double standardization.

# PRE-PROCESSING

As the causes of sensor long-term temporal drift and low overall VOC concentrations are not fully understood, four different data pre-processing pipelines were implemented as shown in Fig. S6.


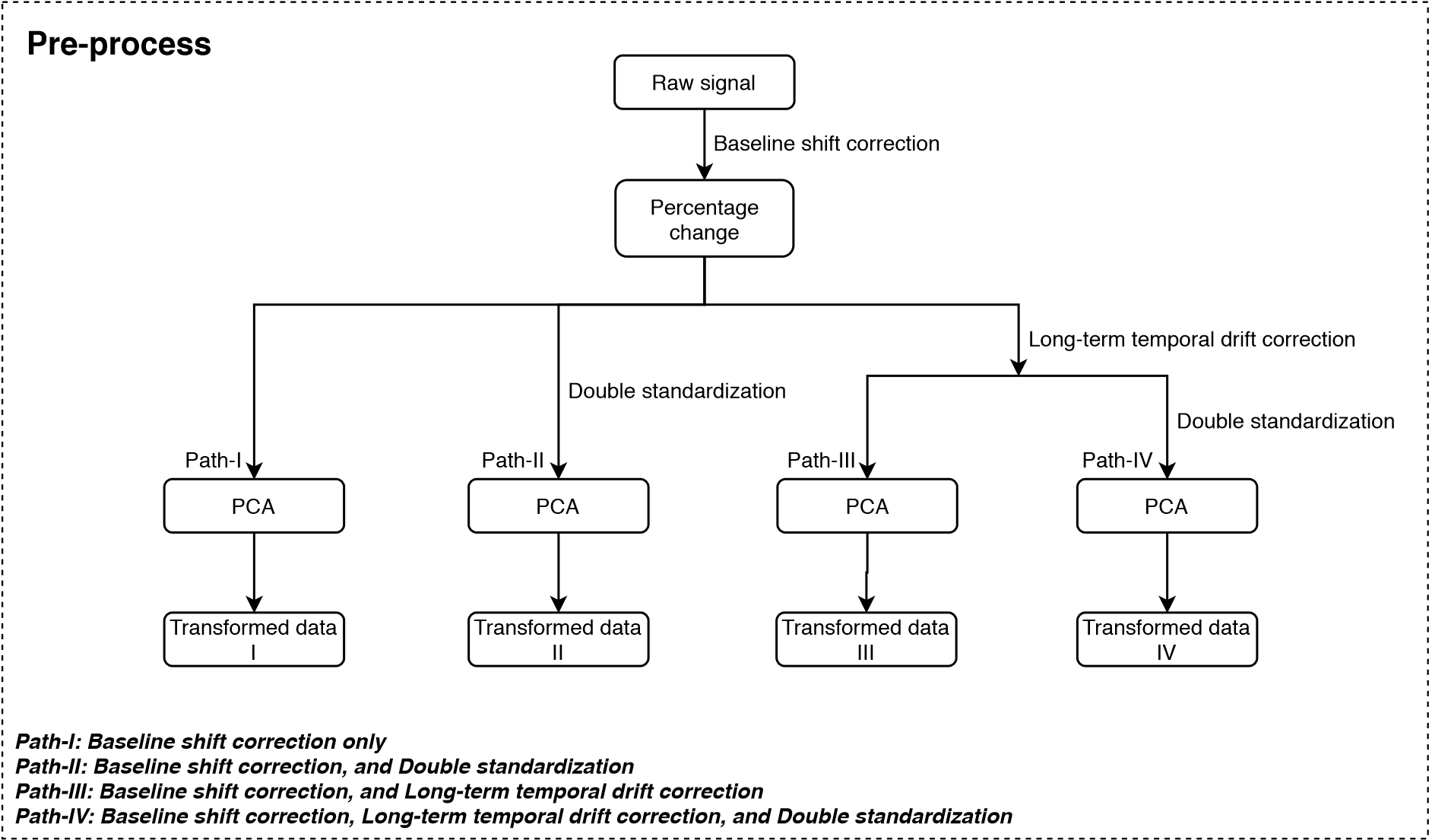


Figure S6. Pre-processing pipeline.

# ADDITIONAL RESULTS

Only results from Path-IV are presented in the main manuscript. In this section, results from other pre-processing paths are presented as well.

## Principal Components Analysis (PCA)

The PCA results from all four pre-processing paths are plotted in Figs. S7, S8 and S9 with respect to sample collection date, gender and smoking status. The first two principal components are strongly related to sample collection date as shown in Figs. S7(A) and S7(B). Long-term temporal drift correction successfully eliminates the time-dependent component in data as shown in Figs. S7(C) and S7(D). However, gender and smoking status are not related with first two principal components as shown in Figs. S8 and S9.


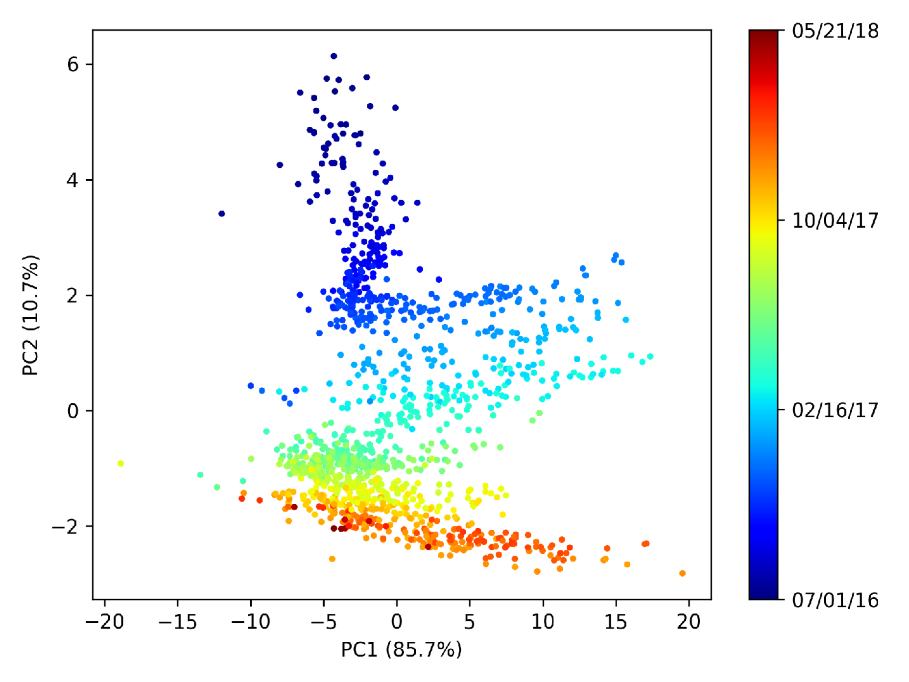

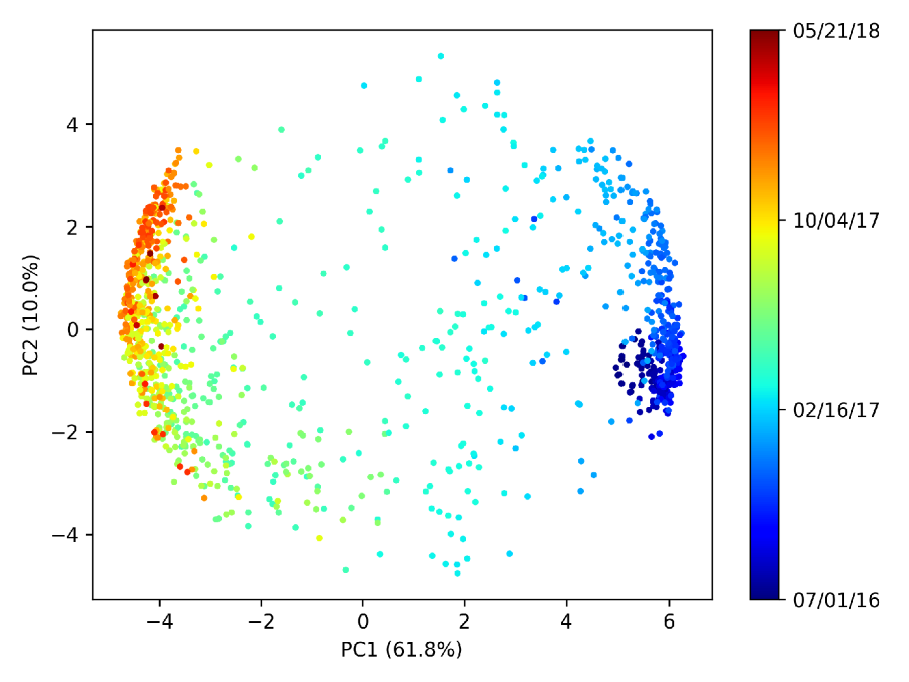


(A) Path-I (B) Path-II


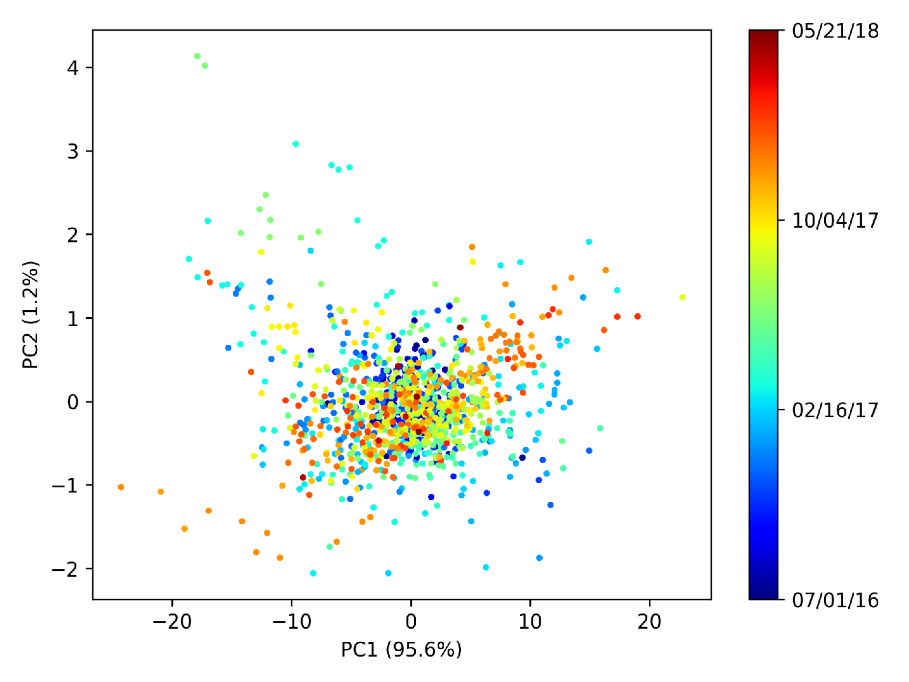

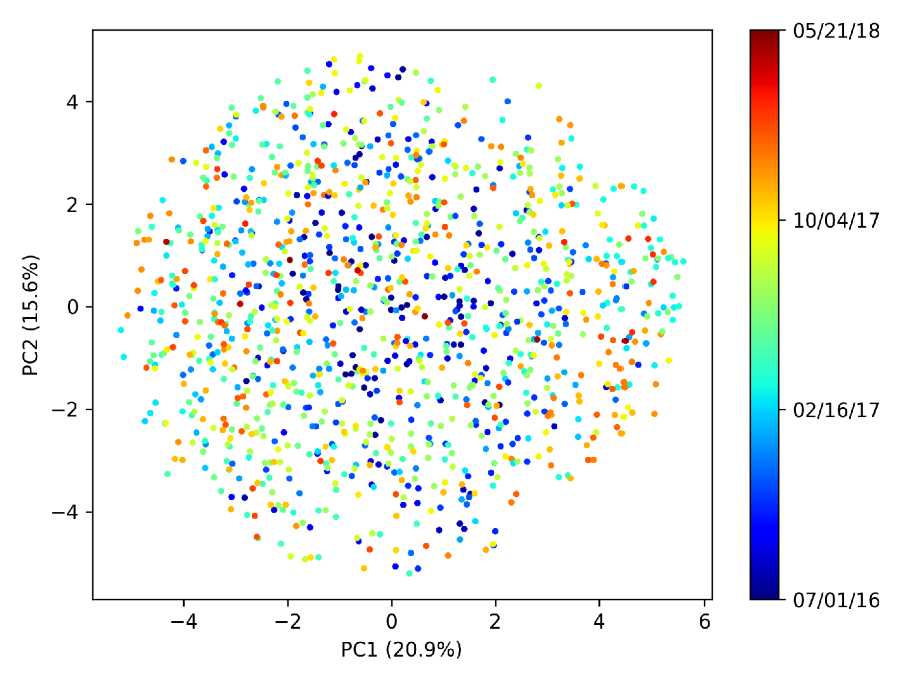


(C) Path-III (D) Path-IV

Figure S7. Principal Components Analysis plot of sample collection date.


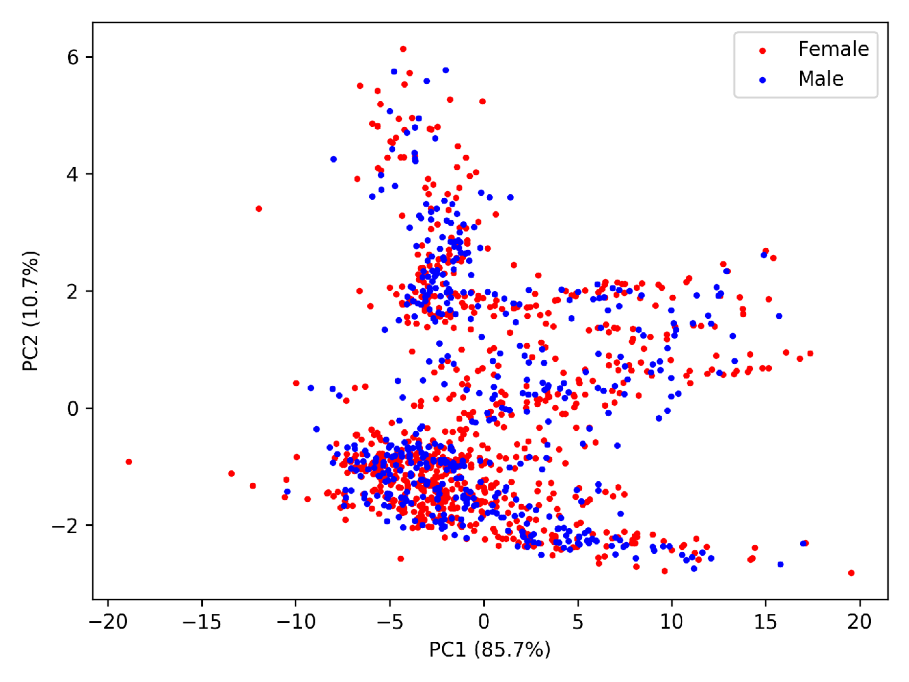

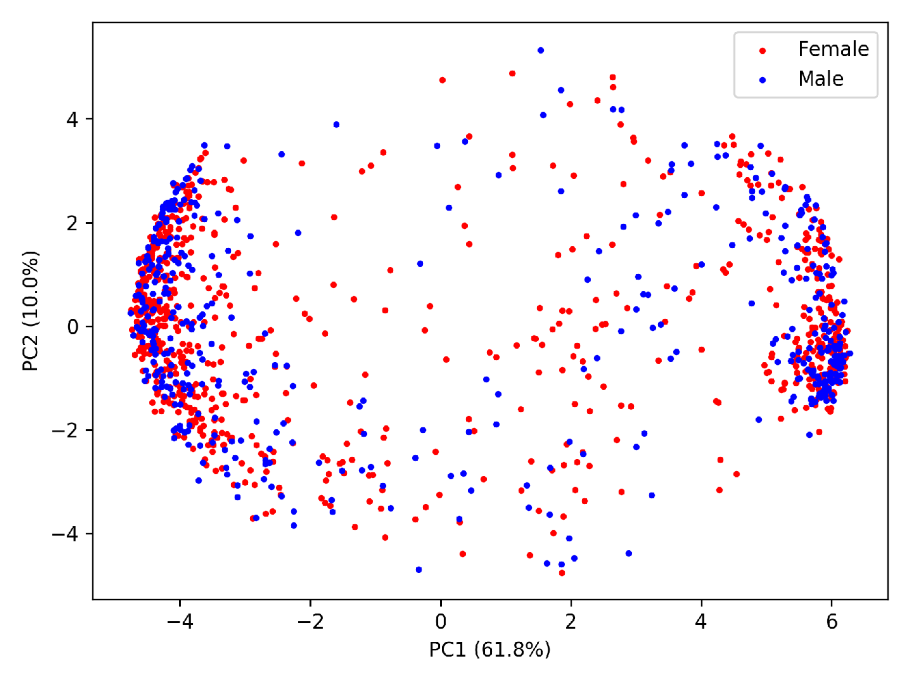


(A) Path-I (B) Path-II


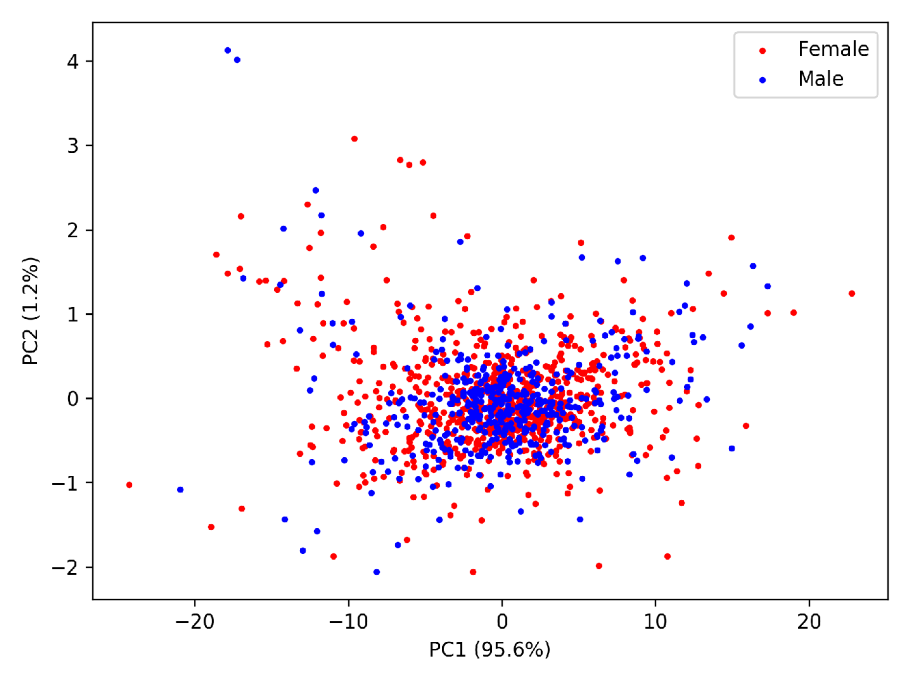

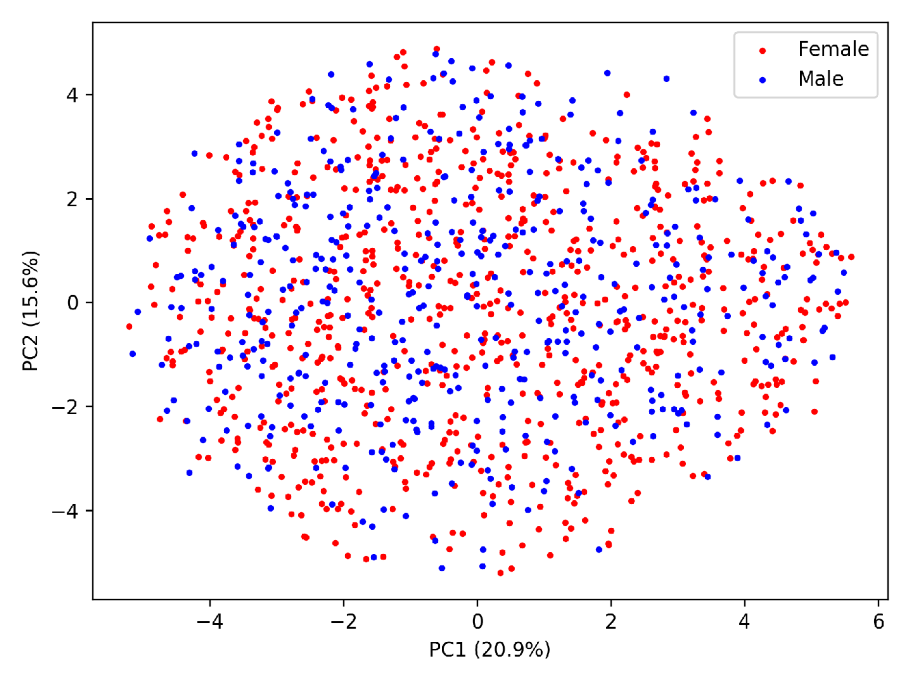


(C) Path-III (D) Path-IV

Figure S8. Principal Components Analysis plot of gender.


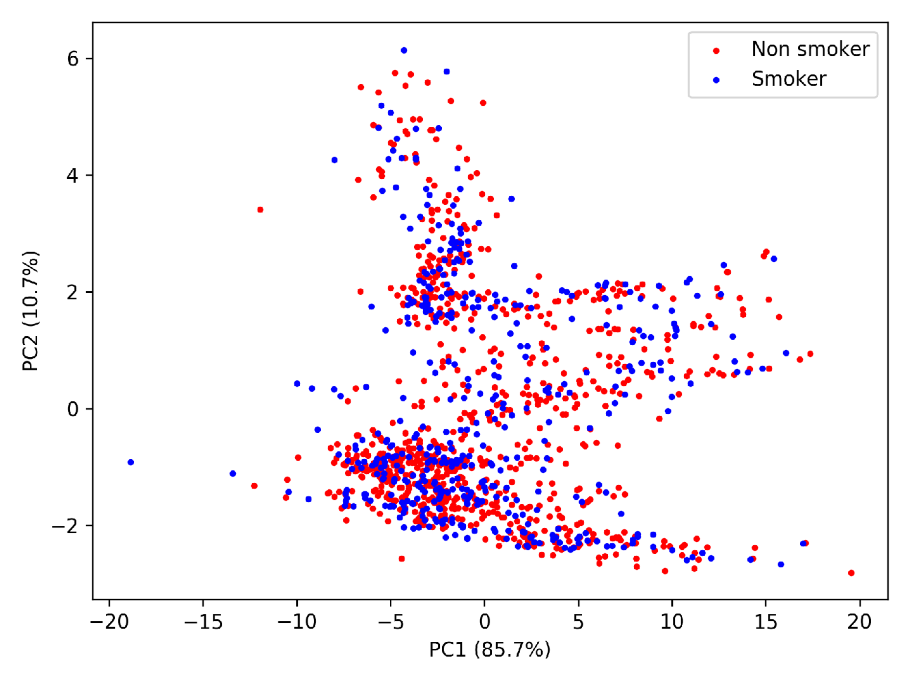

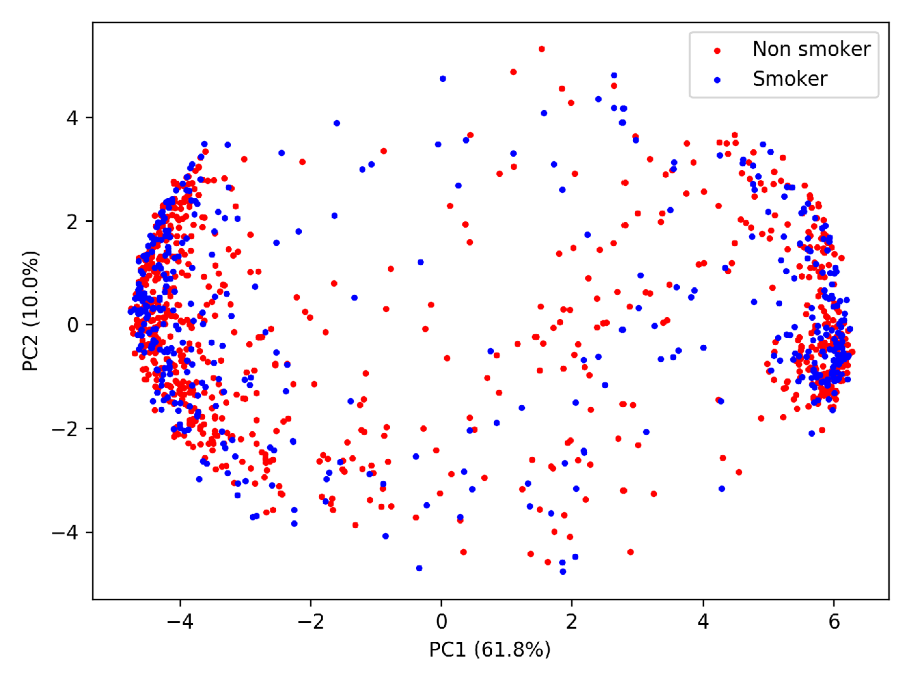


(A) Path-I (B) Path-II


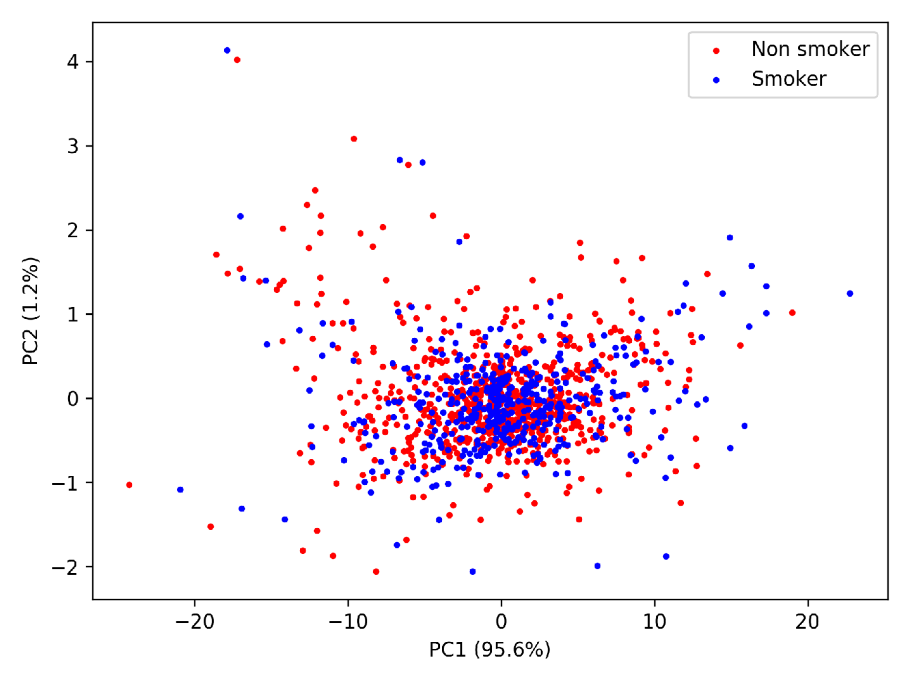

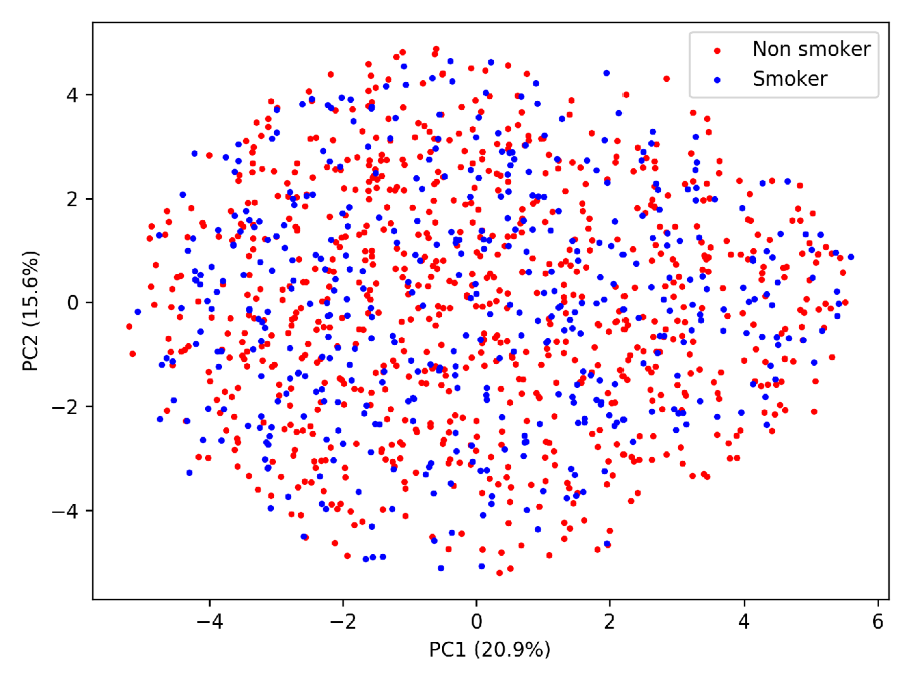


(C) Path-III (D) Path-IV

Figure S9. Principal Components Analysis plot of smoking status.

## Supervised Machine Learning

The results based on machine learning approaches are presented in Figs. S10, S11, S12 and S13. All the results showed poor prediction performance regardless of the pre-processing path used.


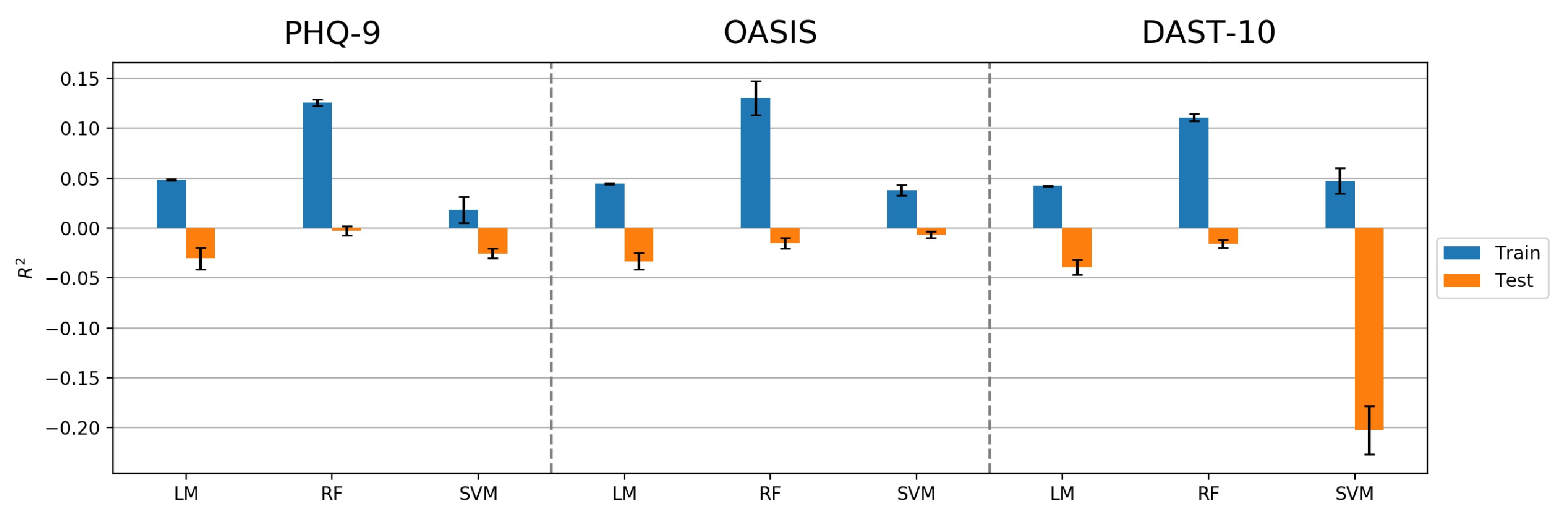


1. *R*^2^ values in predicting PHQ-9, OASIS and DAST-10


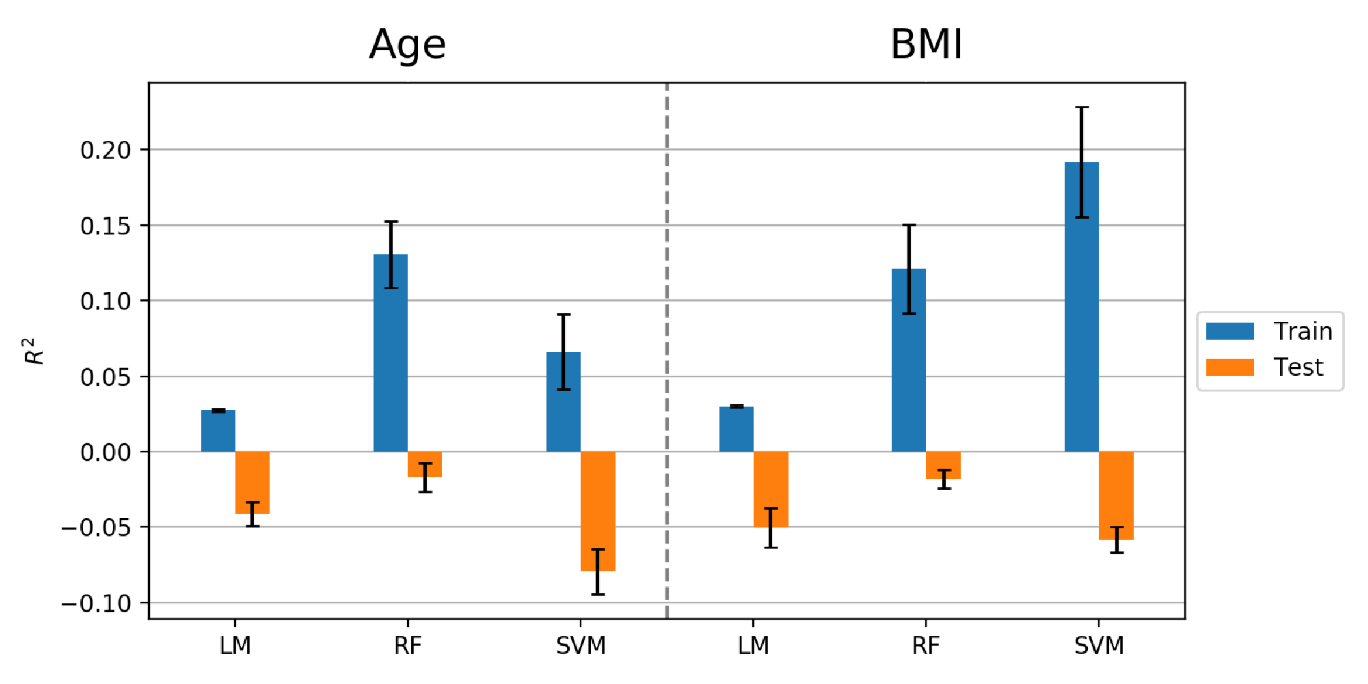


1. *R*^2^ values in predicting age and BMI


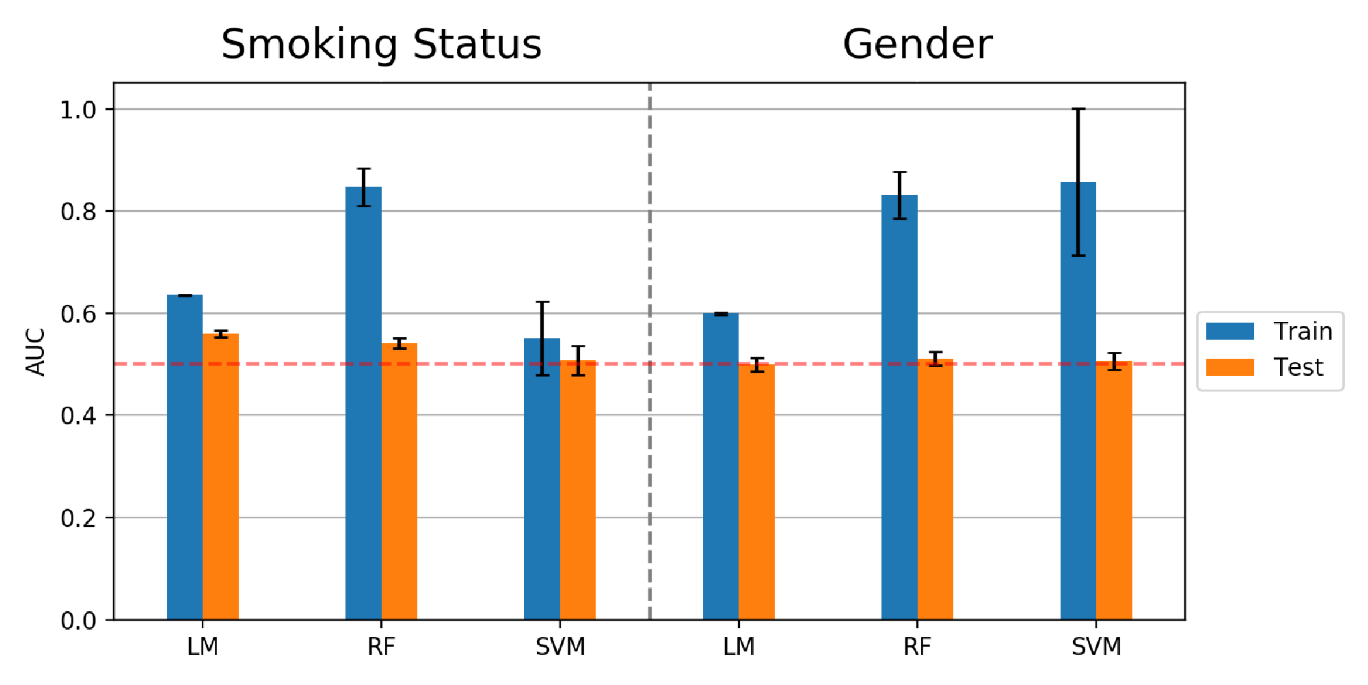


1. AUC values in predicting smoking status and gender

Figure S10. Results of machine learning prediction based on Path-I.


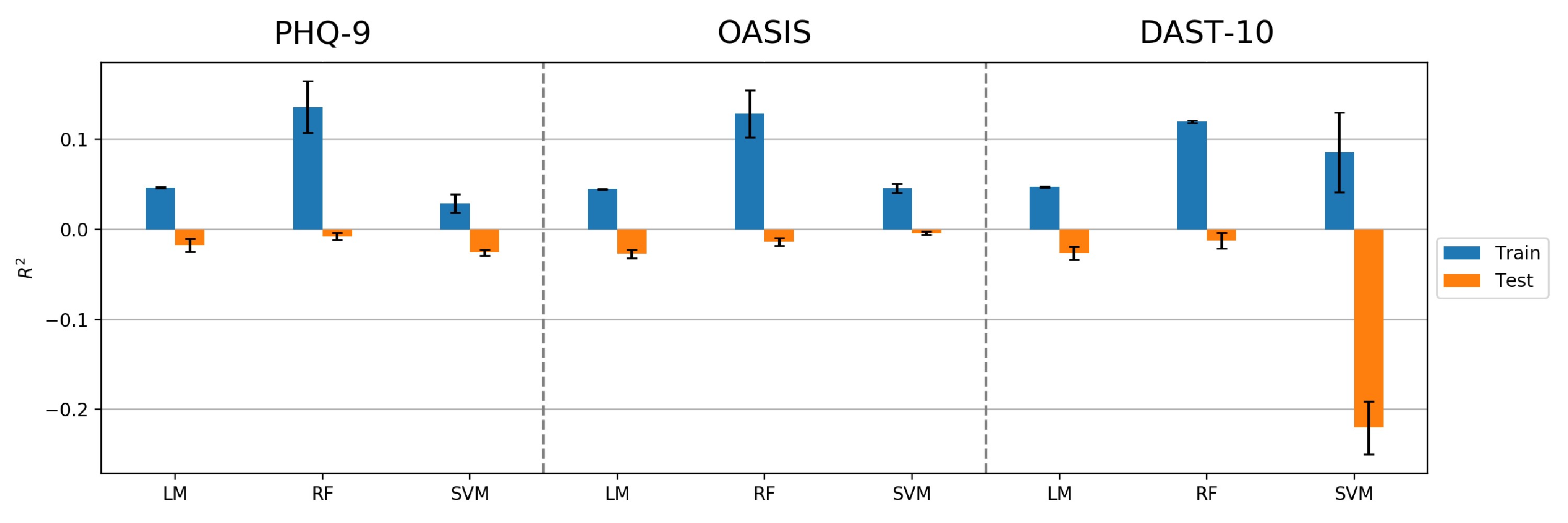


1. *R*^2^ values in predicting PHQ-9, OASIS and DAST-10


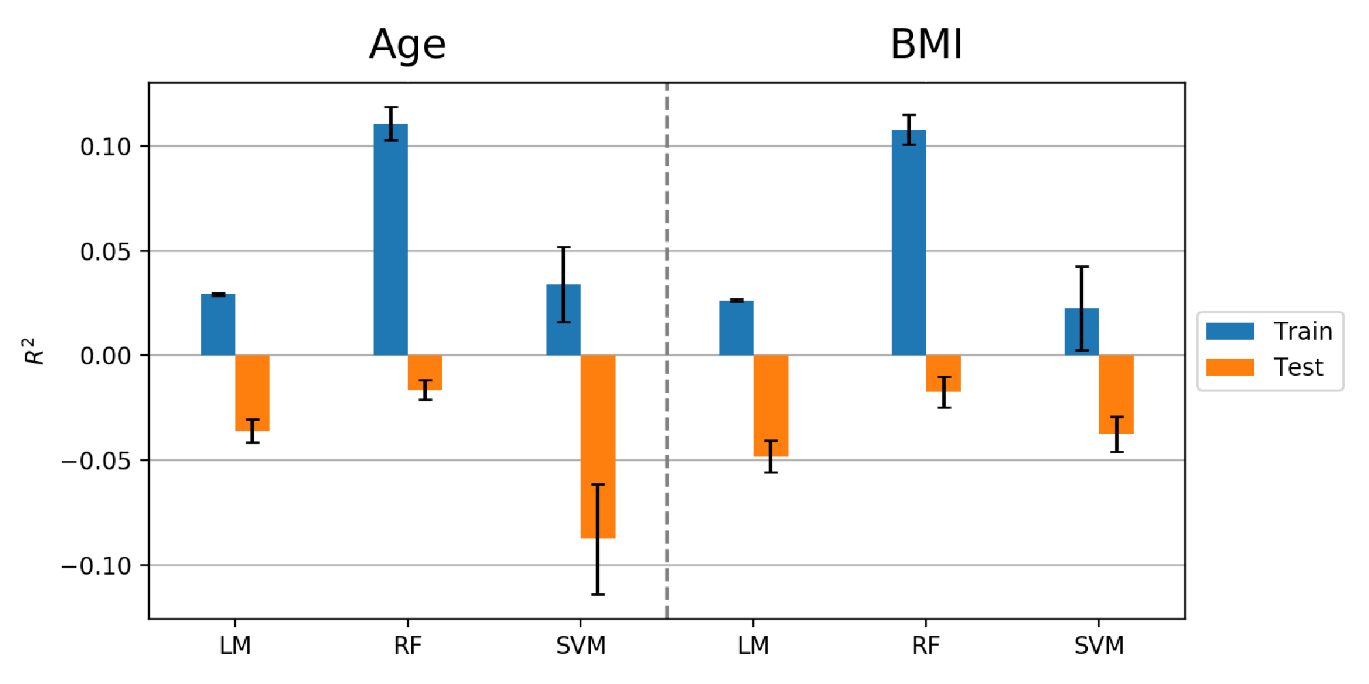


1. *R*^2^ values in predicting age and BMI


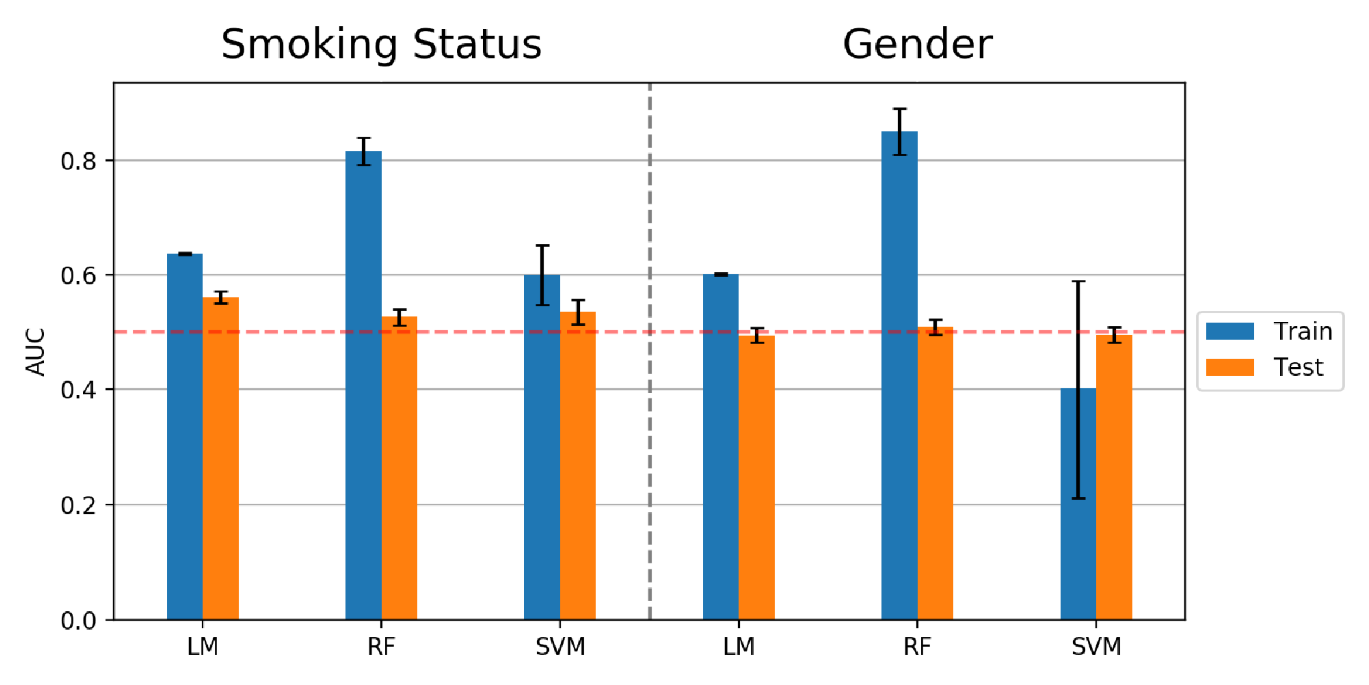


1. AUC values in predicting smoking status and gender

Figure S11. Results of machine learning prediction based on Path-II.


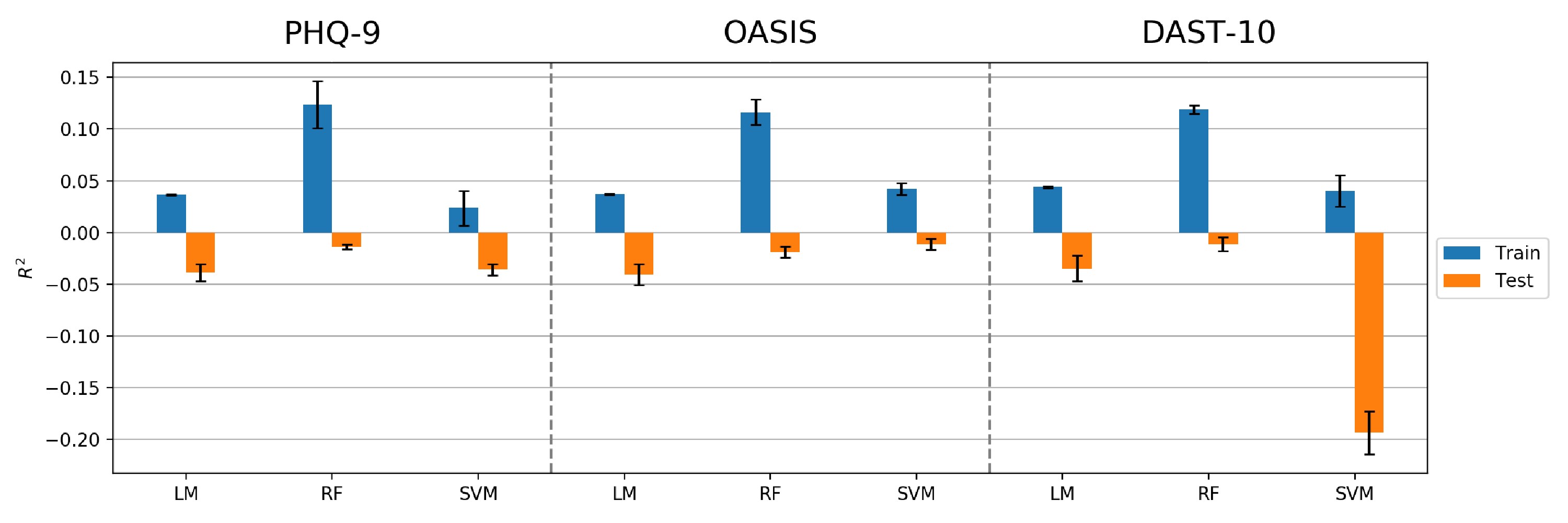


1. *R*^2^ values in predicting PHQ-9, OASIS and DAST-10


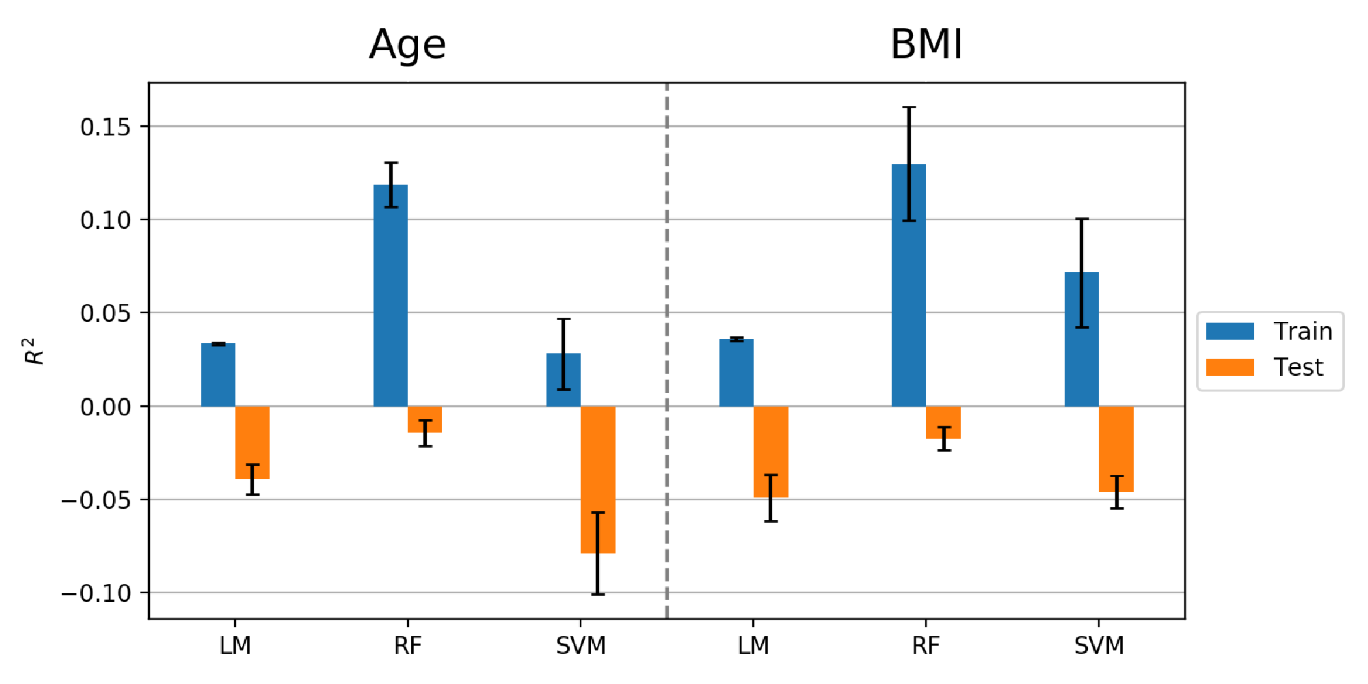


1. *R*^2^ values in predicting age and BMI


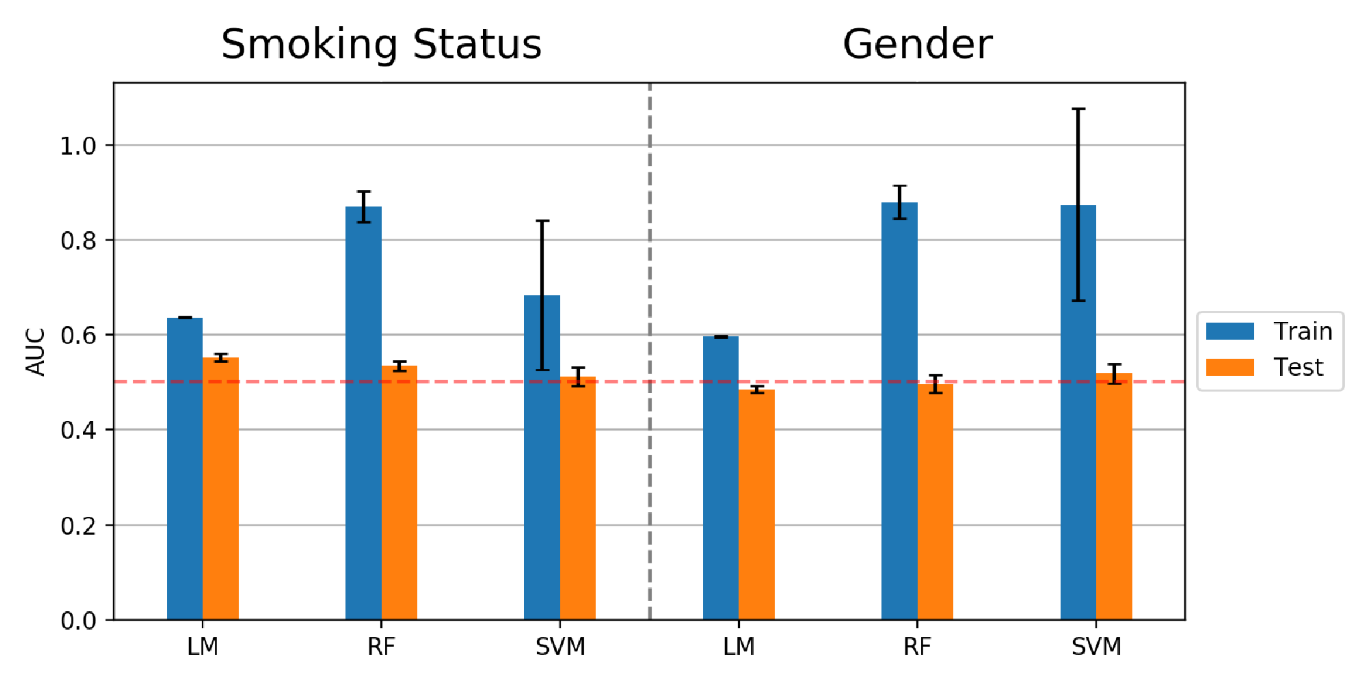


1. AUC values in predicting smoking status and gender

Figure S12. Results of machine learning prediction based on Path-III.


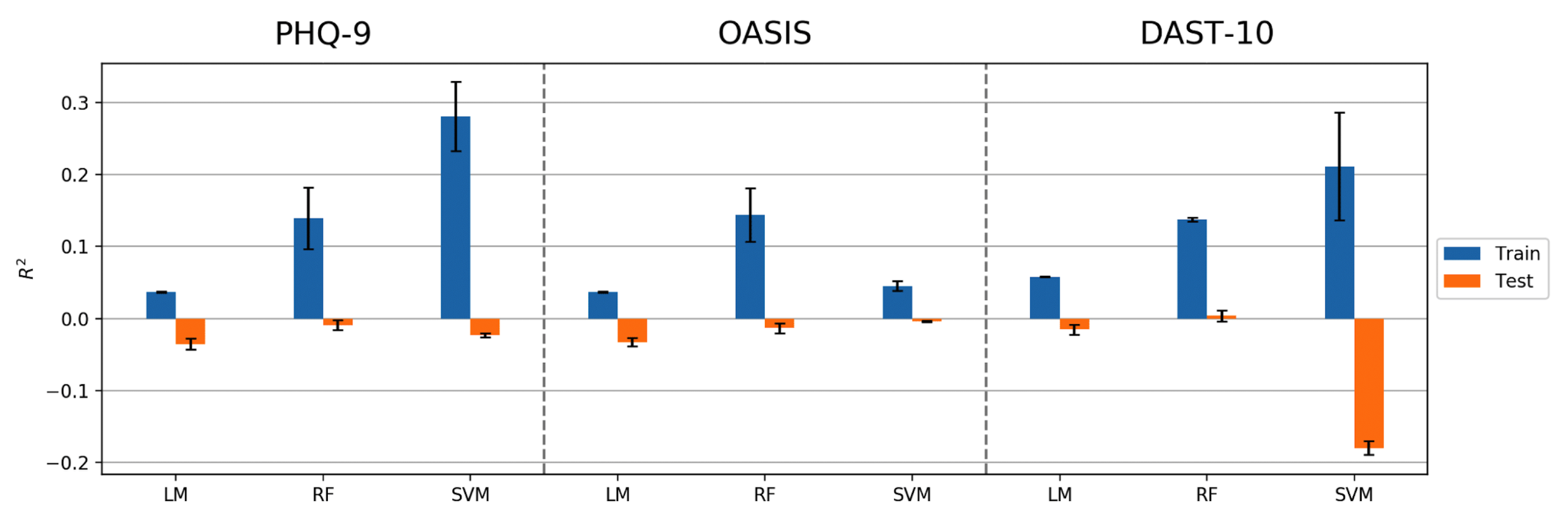


1. *R*^2^ values in predicting PHQ-9, OASIS and DAST-10


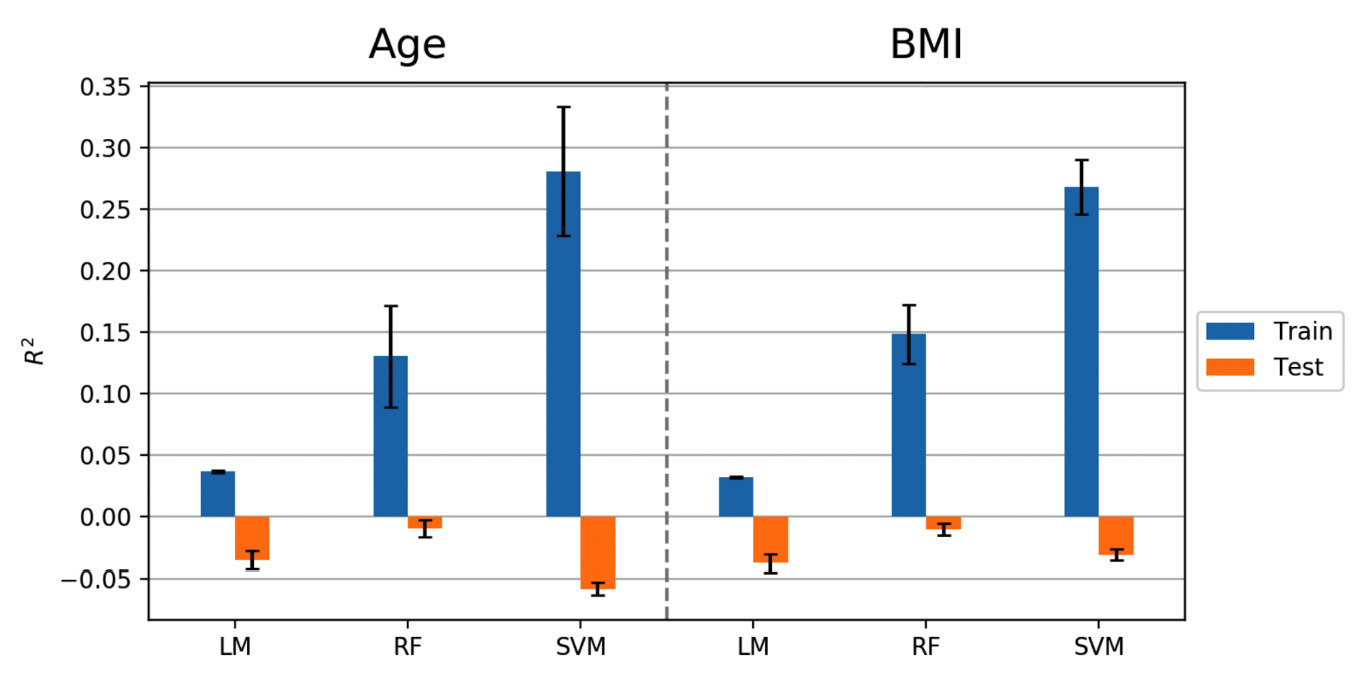


1. *R*^2^ values in predicting age and BMI


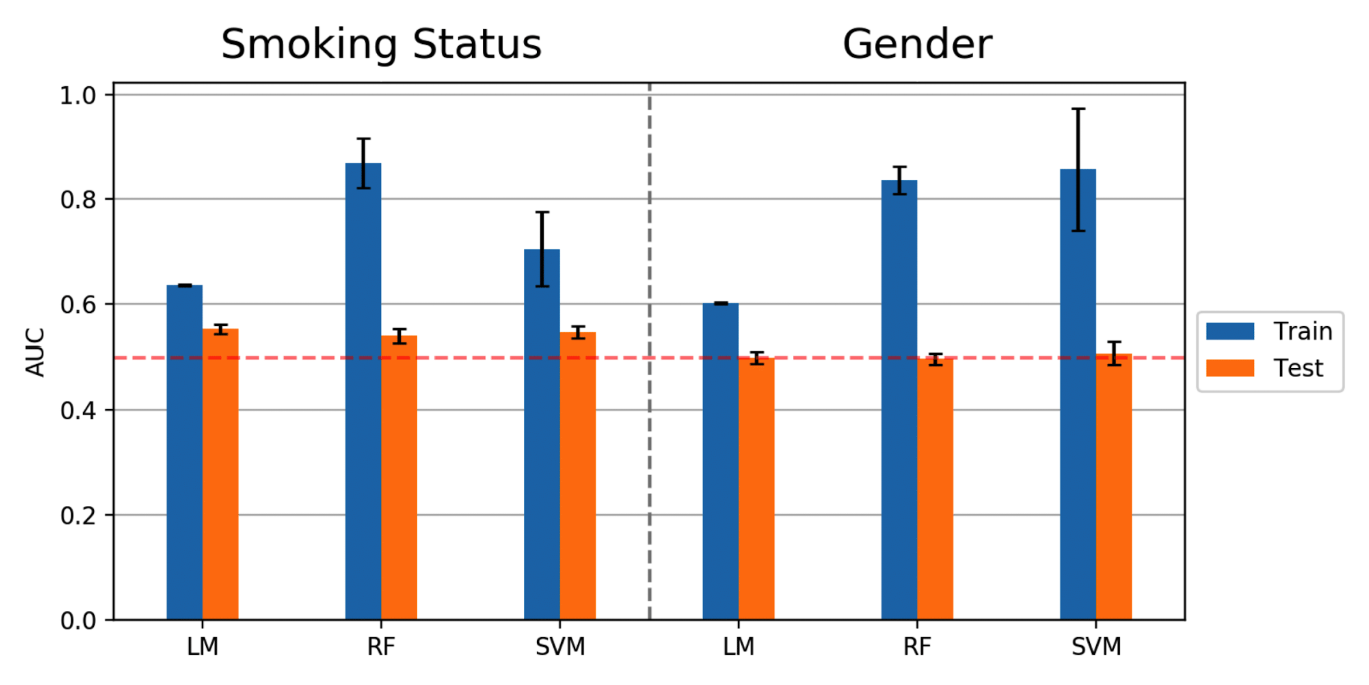


1. AUC values in predicting smoking status and gender

Figure S13. Results of machine learning prediction based on Path-IV.

## More Machine Learning Models and Removal of Water Sensitive Sensors

Elastic net (ENET), Gaussian process (GP) and extreme gradient boosting (XGB) were tested on this dataset. Besides predicting subject mental disorder severity, we also tested whether these algorithms could differentiate subjects into different groups based on Tulsa 1000 thresholds (PHQ-9≥10; OASIS≥8; DAST-10≥3). The corresponding results are listed in Tables S1 and S2.

|  |  | LM | RF | SVM | ENET | GP | XGB |
| --- | --- | --- | --- | --- | --- | --- | --- |
| Age | train | 0.0368 | 0.1307 | 0.2811 | 0.0142 | 1.0000 | 0.2083 |
|  | test | -0.0349 | -0.0093 | -0.0578 | -0.0067 | -0.2071 | -0.0656 |
| BMI | train | 0.0323 | 0.1487 | 0.2682 | 0.0147 | 1.0000 | 0.2118 |
|  | test | -0.0373 | -0.0103 | -0.0306 | -0.0113 | -0.3520 | -0.0558 |
| PHQ-9 | train | 0.0368 | 0.1389 | 0.2809 | 0.0024 | 1.0000 | 0.1958 |
|  | test | -0.0355 | -0.0092 | -0.0231 | -0.0049 | -0.0800 | -0.0793 |
| OASIS | train | 0.0367 | 0.1439 | 0.0454 | 0.0032 | 1.0000 | 0.2037 |
|  | test | -0.0329 | -0.0132 | -0.0041 | -0.0038 | -0.0953 | -0.0593 |
| DAST-10 | train | 0.0579 | 0.1376 | 0.2114 | 0.0143 | 1.0000 | 0.2257 |
|  | test | -0.0154 | 0.0037 | -0.1798 | 0.0006 | -0.0390 | -0.0306 |

Table S1. $R^{2}$ values of prediction for continuous target variables based on all 32 sensors and Path-IV.

|  |  | LM | RF | SVM | ENET | GP | XGB |
| --- | --- | --- | --- | --- | --- | --- | --- |
| Smoking Status | train | 0.6360 | 0.8694 | 0.7051 | 0.5972 | 0.6415 | 0.9867 |
|  | test | 0.5525 | 0.5400 | 0.5464 | 0.5374 | 0.5563 | 0.5102 |
| Gender | train | 0.6014 | 0.8363 | 0.8574 | 0.5472 | 0.8324 | 1.0000 |
|  | test | 0.4994 | 0.4971 | 0.5075 | 0.4899 | 0.5005 | 0.4659 |
| PHQ-9 (classification) | train | 0.5940 | 0.8160 | 0.5590 | 0.5492 | 0.7600 | 1.0000 |
|  | test | 0.4961 | 0.4970 | 0.4986 | 0.5047 | 0.5156 | 0.4894 |
| OASIS (classification) | train | 0.6168 | 0.8880 | 0.5897 | 0.5745 | 0.9979 | 0.9186 |
|  | test | 0.5239 | 0.5143 | 0.4913 | 0.5317 | 0.5133 | 0.5142 |
| DAST-10 (classification) | train | 0.6619 | 0.8733 | 0.7659 | 0.5930 | 0.6481 | 0.9974 |
|  | test | 0.5598 | 0.5638 | 0.5066 | 0.5203 | 0.5290 | 0.5264 |

Table S2. AUC values of prediction for categorical target variables based on all 32 sensors and Path-IV.

As the Sensors 5, 6, 23 and 31 are water sensitive, we repeated our analysis without these four sensors, and the results are listed in Tables S3 and S4.

|  |  | LM | RF | SVM | ENET | GP | XGB |
| --- | --- | --- | --- | --- | --- | --- | --- |
| Age | train | 0.0332 | 0.1199 | 0.2738 | 0.0152 | 1.0000 | 0.1957 |
|  | test | -0.0150 | -0.0023 | -0.0753 | -0.0058 | -0.7277 | -0.0478 |
| BMI | train | 0.0309 | 0.1176 | 0.2083 | 0.0068 | 1.0000 | 0.1848 |
|  | test | -0.0406 | -0.0038 | -0.0355 | -0.0014 | -0.9488 | -0.0559 |
| PHQ-9 | train | 0.0347 | 0.1174 | 0.0657 | 0.0094 | 1.0000 | 0.1954 |
|  | test | -0.0464 | -0.0246 | -0.0311 | -0.0092 | -0.3764 | -0.0811 |
| OASIS | train | 0.0337 | 0.1025 | 0.0615 | 0.0004 | 1.0000 | 0.1980 |
|  | test | -0.0342 | -0.0201 | -0.0045 | -0.0016 | -0.4517 | -0.0796 |
| DAST-10 | train | 0.0506 | 0.1340 | 0.2736 | 0.0100 | 1.0000 | 0.2246 |
|  | test | -0.0114 | -0.0094 | -0.1883 | 0.0040 | -0.1479 | -0.0513 |

Table S3. $R^{2}$ values of prediction for continuous target variables based on 28 sensors and Path-IV.

|  |  | LM | RF | SVM | ENET | GP | XGB |
| --- | --- | --- | --- | --- | --- | --- | --- |
| Smoking Status | train | 0.6287 | 0.8857 | 0.6773 | 0.5834 | 0.6389 | 0.8415 |
|  | test | 0.5540 | 0.5342 | 0.5428 | 0.5510 | 0.5582 | 0.5577 |
| Gender | train | 0.6041 | 0.8877 | 0.8711 | 0.5461 | 0.6041 | 1.0000 |
|  | test | 0.4818 | 0.4875 | 0.4836 | 0.4954 | 0.4828 | 0.4978 |
| PHQ-9 (classification) | train | 0.5959 | 0.8034 | 0.3941 | 0.5653 | 0.8325 | 0.9714 |
|  | test | 0.5121 | 0.5236 | 0.4822 | 0.4828 | 0.5278 | 0.5159 |
| OASIS (classification) | train | 0.6094 | 0.8294 | 0.5657 | 0.5552 | 0.9250 | 0.9774 |
|  | test | 0.5292 | 0.5148 | 0.5026 | 0.5206 | 0.5373 | 0.4962 |
| DAST-10 (classification) | train | 0.6490 | 0.8431 | 0.4221 | 0.5971 | 0.6676 | 0.9104 |
|  | test | 0.5547 | 0.5384 | 0.5114 | 0.5625 | 0.5646 | 0.5558 |

Table S4. AUC values of prediction for categorical target variables based on 28 sensors and Path-IV.

Based on the $R^{2}$ and AUC values in these four tables, all these models can account for some variance in the training sets, but the results do not generalize to the test sets. Although not depicted here, the other three pre-processing paths provided similar results as Path-IV.

## Diurnal Variations of Cyranose 320 Sensor Responses

All the samples were collected between 8:00 AM and 6:30 PM. However, we did not observe any diurnal “smellprint” variation during our sample collection period. We used Sensor 1 as an example and plot sample collection time in a day vs sensor response. All other sensor responses show a similar pattern to Sensor 1.


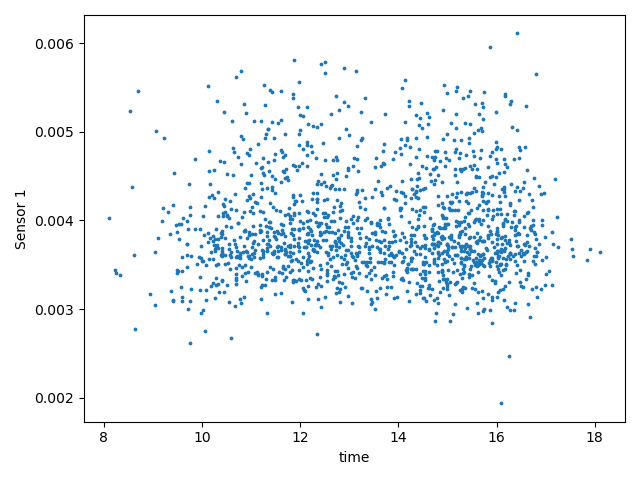


Figure S14. Diurnal effects on Sensor 1 responses between 8:00 am and 6:30 pm.

# REFERENCES

Bikov A, Lázár Z, Horvath I. Established methodological issues in electronic nose research: how far are we from using these instruments in clinical settings of breath analysis? *Journal of breath research* 9 (2015) 034001.

James D, Scott SM, Ali Z, O’hare WT. Chemical sensors for electronic nose systems. *Microchimica Acta* 149 (2005) 1–17.
